# Crystallographic and electrophilic fragment screening of the SARS-CoV-2 main protease

**DOI:** 10.1038/s41467-020-18709-w

**Published:** 2020-10-07

**Authors:** Alice Douangamath, Daren Fearon, Paul Gehrtz, Tobias Krojer, Petra Lukacik, C. David Owen, Efrat Resnick, Claire Strain-Damerell, Anthony Aimon, Péter Ábrányi-Balogh, José Brandão-Neto, Anna Carbery, Gemma Davison, Alexandre Dias, Thomas D. Downes, Louise Dunnett, Michael Fairhead, James D. Firth, S. Paul Jones, Aaron Keeley, György M. Keserü, Hanna F. Klein, Mathew P. Martin, Martin E. M. Noble, Peter O’Brien, Ailsa Powell, Rambabu N. Reddi, Rachael Skyner, Matthew Snee, Michael J. Waring, Conor Wild, Nir London, Frank von Delft, Martin A. Walsh

**Affiliations:** 1 Diamond Light Source Ltd., Harwell Science and Innovation Campus, Didcot, OX11 0QX UK; 2 Research Complex at Harwell, Harwell Science and Innovation Campus, Didcot, OX11 0FA UK; 3 Department of Organic Chemistry, Weizmann Institute of Science, Rehovot, 7610001 Israel; 4 Structural Genomics Consortium, University of Oxford, Old Road Campus, Roosevelt Drive Headington, OX3 7DQ UK; 5 Medicinal Chemistry Research Group, Research Centre for Natural Sciences, Magyar tudósok krt. 2, H-1117 Budapest, Hungary; 6 Department of Statistics, University of Oxford, Oxford, OX1 3LB UK; 7 Cancer Research UK Drug Discovery Unit, Newcastle University Centre for Cancer, Chemistry, School of Natural and Environmental Sciences, Bedson Building, Newcastle University, Newcastle upon Tyne, NE1 7RU UK; 8 Department of Chemistry, University of York, Heslington York, YO10 5DD UK; 9 Cancer Research UK Drug Discovery Unit, Newcastle University Centre for Cancer, Paul O’Gorman Building, Medical School, Framlington Place, Newcastle University, Newcastle upon Tyne, NE2 4AD UK; 10 Department of Biochemistry, University of Johannesburg, Auckland Park, 2006 South Africa

**Keywords:** Enzymes, Proteases, Proteases, X-ray crystallography, Mass spectrometry

## Abstract

COVID-19, caused by SARS-CoV-2, lacks effective therapeutics. Additionally, no antiviral drugs or vaccines were developed against the closely related coronavirus, SARS-CoV-1 or MERS-CoV, despite previous zoonotic outbreaks. To identify starting points for such therapeutics, we performed a large-scale screen of electrophile and non-covalent fragments through a combined mass spectrometry and X-ray approach against the SARS-CoV-2 main protease, one of two cysteine viral proteases essential for viral replication. Our crystallographic screen identified 71 hits that span the entire active site, as well as 3 hits at the dimer interface. These structures reveal routes to rapidly develop more potent inhibitors through merging of covalent and non-covalent fragment hits; one series of low-reactivity, tractable covalent fragments were progressed to discover improved binders. These combined hits offer unprecedented structural and reactivity information for on-going structure-based drug design against SARS-CoV-2 main protease.

## Abstract (web summary)

The SARS-CoV-2 main protease is an important target for the development of COVID-19 therapeutics. Here, the authors combine X-ray crystallography and mass spectrometry and performed a large scale fragment screening campaign, which yielded 96 liganded structures of this essential viral protein that are of interest for further drug development efforts.

## Introduction

A novel coronavirus, SARS-CoV-2, the causative agent of COVID-19^1–3^, has resulted in over one million confirmed cases and in excess of 300,000 deaths across 188 countries as of mid-May 2020^4^. SARS-CoV-2 is the third zoonotic coronavirus outbreak after the emergence of SARS-CoV-1 in 2002 and the Middle East Respiratory Syndrome (MERS-CoV) in 2012^5–7^. SARS-CoV-2 is a large enveloped, positive-sense, single-stranded RNA Betacoronavirus. The viral RNA encodes two open reading frames that, through ribosome frame-shifting, generates two polyproteins pp1a and pp1ab^8^. These polyproteins produce most of the proteins of the replicase-transcriptase complex^9^. The polyproteins are processed by two viral cysteine proteases: a papain-like protease (PL^pro^) which cleaves three sites, releasing non-structural proteins nsp1-3 and a 3C-like protease, also referred to as the main protease (M^pro^), that cleaves at 11 sites to release non-structural proteins (nsp4-16). These non-structural proteins form the replicase complex responsible for replication and transcription of the viral genome and have led to M^pro^ and PL^Pro^ being the primary targets for antiviral drug development^10^.

Structural biology, which can play a key role in drug development, was also rapidly deployed after the 2002 SARS-CoV-1 outbreak, with earlier work by the Hilgenfeld group on M^pro^ of coronarviruses^10^ leading to crystal structures of SARS-CoV-1 M^pro^ and inhibitor complexes^11–14^. Active sites of Coronavirus M^pro^ are well conserved^13,15–19^, and those of enteroviruses (3C^pro^) are functionally similar: this underpins ambitions to develop broad-spectrum antivirals. The most successful have been peptidomimetic α-ketoamide inhibitors^20^, with at least one potent variant seen as a potential antiviral drug^19^. Other studies have taken the popular approach of high-throughput screens (HTS) using very large compound libraries, followed by structural studies to elucidate the binding mode^21^.

Despite these efforts, drugs remain elusive that directly target SARS-CoV-2 (rather than disease symptoms) and are verified by clinical trials. In retrospect, this is perhaps unsurprising for the M^pro^ inhibitors, as both peptidomimetic and covalent inhibition carry risks as strategies for drug development; in general, the simpler the molecule, the lower the risk.

We therefore applied a different approach to M^pro^, using fragment screening by high-throughput structural biology^22^. Fragment methods have become a staple of modern drug discovery^23^, using small collections (100 s or 1000 s) of small compounds (<300 Da) that bind promiscuously and thus sample a far larger chemical space than is achieved by HTS. The challenge is that the very weak binding of fragment hits necessitates highly sensitive biophysical detection, careful confirmation of binding and specialised medicinal chemistry expertise to advance hits to potency. Their promise is that potency can be achieved with high efficiency, simplifying the progression of molecules to biological or clinical impact.

While the screening experiment itself has long relied on the high throughput of solution methods like NMR or SPR^23^, rapid advances in technology and automation at synchrotron radiation sources^24^ has made screening directly in crystal structures routinely possible at facilities like the XChem platform at Diamond Light Source^25–28^. These have been further enhanced by techniques such as mass spectrometry for the discovery of covalently binding fragments^29^.

In the current study, we screened M^pro^ of SARS-CoV-2 with over 1250 unique fragments, identifying 74 high-value fragment hits, including 23 non-covalent and 48 covalent hits in the active site, and 3 hits at the vital dimerization interface. Here, these data are detailed along with potential ways forward for rapid follow-up design of improved, more potent, compounds.

## Results

### M^pro^ crystallizes in a ligand-free form that diffracts to near-atomic resolution

**Fig. 1:**
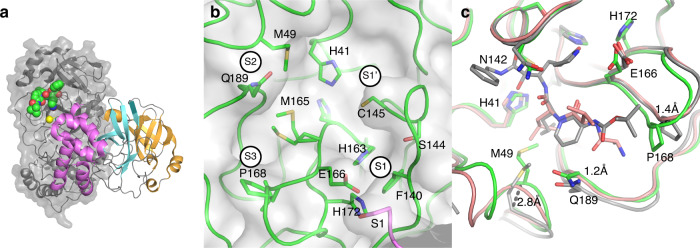
The crystal structure of ligand free M^pro^ is amenable to X-ray fragment screening. **a** Cartoon representation of the M^pro^ dimer. The nearmost monomer is shown with secondary structure features coloured to demarcate domains I, II, and III, in orange, cyan, and violet respectively. The active site of the rear monomer is indicated by the presence of a peptide-based inhibitor in green, generated by aligning the ligand-free structure with pdb 6Y2F [10.2210/pdb6y2f/pdb]. A yellow sphere indicates Ser1 from the dimer partner that completes the active site. **b** Residues of the active site are labelled, and subsites involved in ligand binding are shown with circles. **c** Active site plasticity is observed when comparing the apo structure to peptide inhibitor bound structures (green—Apo, grey—6Y2F [10.2210/pdb6y2f/pdb], pink 6LU7 [10.2210/pdb6lu7/pdb]). Displacement distances associated with loop movements are indicated.

We report the apo structure of SARS-CoV-2 M^pro^ with data to 1.25 Å. The construct we crystallised has native residues at both N- and C--terminals, without cloning truncations or appendages which could otherwise interfere with fragment binding. Electron density is present for all residues, including 26 alternate conformations, many of which were absent in previous lower resolution crystal structures. The protein crystallised with a single protein polypeptide in the asymmetric unit, and the catalytic dimer is provided by a symmetry-related molecule. The structure aligns closely with the M^pro^ structures from SARS-CoV-1 and MERS (rmsd of 0.52 Å and 0.97 Å respectively). The active site is sandwiched between two β-barrel domains, I (residue 10–99) and II (residue 100–182) (Fig. 1a). Domain III (residue 198–306), forms a bundle of alpha helices and is proposed to regulate dimerization^30^. The C-terminal residues, Cys300-Gln306, wrap against Domain II. However, the C terminal displays a degree of flexibility and wraps around domain III in the N3 inhibitor complex^30^ (PDB ID 6LU7 [10.2210/pdb6lu7/pdb]). His41 and Cys145 comprise the catalytic dyad and dimerisation completes the active site by bringing Ser1 of the second dimer protomer into proximity with Glu166 (Fig. 1b). This aids formation of the substrate specificity pocket and the oxyanion hole^10^. Subsites have previously been identified in the active site based on interactions with peptide-based inhibitors and are shown in Fig. 1b^19,31^. Comparisons with peptide-based inhibitor complexes^19,31^ suggest a degree of active site plasticity. In particular, the C-alphas of Met49, Pro168, Gln189 respectively show movements of 2.8 Å, 1.4 Å, and 1.2 Å in comparison to the α-ketoamide inhibitor bound M^pro^ structure^19^ (PDB ID 6y2f [10.2210/pdb6y2f/pdb], Fig. 1c).

The crystal form is well-suited for crystallographic fragment screening: although the percentage of solvent (~20%) is very low for a protein crystal, nevertheless clear channels are present that allow access to the active site through diffusion. Moreover, the tight packing and strong innate diffraction mean crystals are resistant to lattice disruption and degradation of diffraction by DMSO solvent when adding solubilised fragments to the crystallization drop.

### Combined MS and crystallographic fragment screens reveal new binders of M^pro^

Cysteine proteases are attractive targets for covalent inhibitors, and screening covalent fragments is known to be useful at identifying effective starting points^32–36^. To identify covalent starting points, we screened our previously described library of ~1000 mild electrophilic fragments^29^ against M^pro^ using intact protein mass spectrometry. Standard conditions of 200 µM per electrophile for 24 h at 4 °C did not allow discrimination between hits. Screening at more stringent conditions (5 µM per electrophile; 1.5 h; 25 °C) resulted in 8.5% of the library labelling above 30% of protein (Supplementary Data 1). These hits revealed common motifs, and we focused on compounds that offer promising starting points.

Compounds containing *N*-chloroacetyl-*N´*-sulfonamido-piperazine or *N-*chloroacetylaniline motifs were frequent hitters. Such compounds can be highly reactive. Therefore, we chose series members with relatively low reactivity for follow-up crystallization attempts. For another series of hit compounds, containing a *N*-chloroacetyl piperidinyl-4-carboxamide motif (Supplementary Data 1) which displays lower reactivity and were not frequent hitters in previous screens, we attempted crystallization despite their absence of labelling in the stringent conditions.

**Fig. 2:**
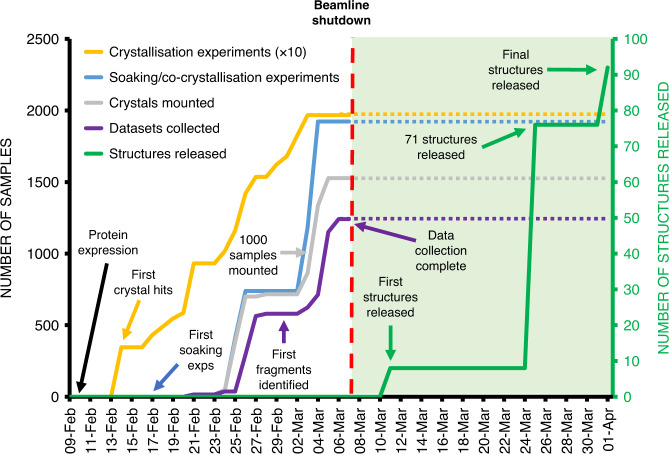
Timeline of crystallographic fragment screen. Progress of the Mpro fragment screening experiment from the start of protein production and purification (9 Feb 2020) to the deposition and release of the high-resolution ligand-free structure of Mpro PDB ID 6YB7[10.2210/pdb6yb7/pdb] and the structures of the 96 fragment hits identified in the fragment screening campaign using the XChem platform at Diamond Light Source.

While mild electrophilic fragments are ideal for probing the binding properties around the active site cysteine, their small size prevents extensive exploration of the substrate-binding pocket. We performed an additional crystallographic fragment screen to exhaustively probe the M^pro^ active site, and to find opportunities for fragment merging or growing. The 68 electrophile fragment hits were added to crystals along with a total of 1176 unique fragments from 7 libraries (Supplementary Table 1). Non-covalent fragments were soaked^26^, whereas electrophile fragments were both soaked and co-crystallized as previously described^29^, to ensure that as many of the mass-spectrometry hits as possible were structurally observed. A total of 1742 soaking and 1139 co-crystallization experiments resulted in 1877 mounted crystals. While some fragments either destroyed the crystals or their diffraction, 1638 datasets with a resolution better than 2.8 Å were collected. The best crystals diffracted to better than 1.4 Å, but diffraction to 1.8 Å was more typical, and no datasets worse than 2.8 Å were included in analysis (Supplementary Fig. 2). We identified 96 fragment hits using the PanDDA method^37^, all of which were deposited in the Protein Data Bank (Supplementary Data 2), but also immediately released through the Diamond Light Source website (https://www.diamond.ac.uk/covid-19.html), along with all protocols and experimental details. A timeline of experiments is shown in Fig. 2.

### Non-covalent fragment hits reveal multiple targetable sub-sites in the active site

This unusually large screen identified 23 structurally diverse fragments that bind non-covalently and extensively sample features of the M^pro^ active site and its specificity pockets/subsites (Fig. 1), along with three hits exploring the dimer interface.

**Fig. 3:**
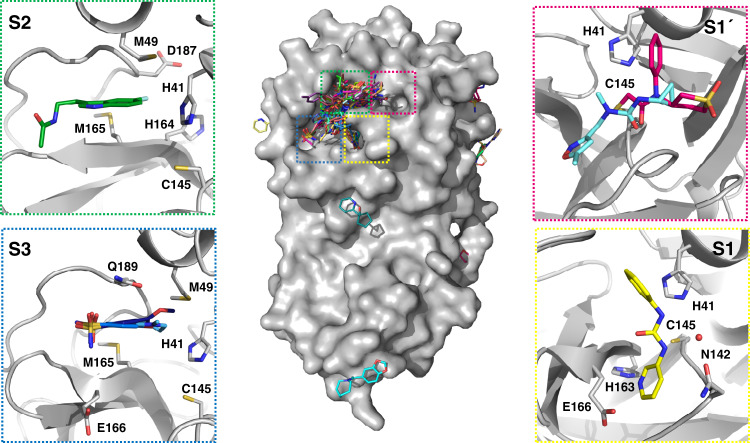
Bound fragments sample the active site comprehensively. The central surface representation is of the M^pro^ monomer with all fragment hits shown as sticks, and active site subsites highlighted by coloured boxes. Each subsite is expanded along with a selection of hits to demonstrate common features and interactions. **S1**: Z44592329 (x0434); **S1**′: Z369936976 (×0397) in aquamarine and PCM-0102372 (×1311) in magenta bound to active site cysteine; **S2**: Z1220452176 (x0104); **S3**: Overlay of Z18197050 (×0161), Z1367324110 (×0195) and NCL-00023830 (×0946).

Eight fragments were identified that bind in the S1 subsite and frequently form interactions with the side chains of the key residues His163, through a pyridine ring or similar nitrogen-containing heterocycle, and Glu166 through a carbonyl group in an amide or urea moiety (Fig. 3). Several also reach across into the S2 subsite. Subsite S2 has previously demonstrated greater flexibility in comparison to the other subsites, adapting to smaller substituents in peptide-based inhibitors but with a preference for leucine or other hydrophobic residues^19^. Many fragments bound at this location, which we termed the “aromatic wheel” because of a consistent motif of an aromatic ring forming hydrophobic interactions with Met49 or π–π stacking with His41, with groups variously placed in 4 axial directions. Particularly notable is the vector into the small pocket between His164, Met165 and Asp187, exploited by three of the fragments (Z1220452176 (x0104), Z219104216 (x0305) and Z509756472 (×1249)) with fluoro and cyano substituents (Fig. 3).

Of the four fragments exploring subsite S3, three contain an aromatic ring with a sulfonamide group forming hydrogen bonds with Gln189 and pointing out of the active site towards the solvent interface (Fig. 3). These hits have expansion vectors suitable for exploiting the same His164/Met165/Asp187 pocket mentioned above.

**Fig. 4:**
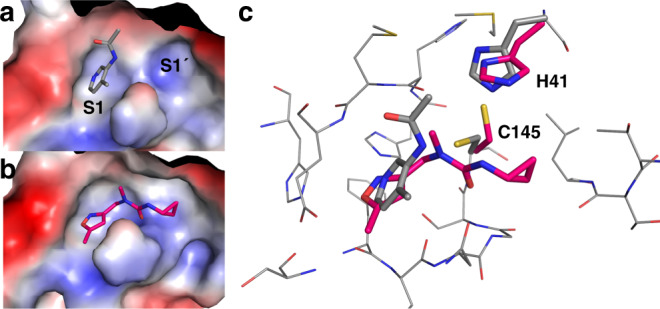
Plasticity of S1´ is revealed by fragment Z369936976 (×0397). Comparing the electrostatic surfaces of Z1129283193 (×0107) **a** The most commonly observed conformation, with that of Z369936976 (x0397). **b** How the shape of S1 and S1´ can change. **c** Sidechain movement of catalytic residues Cys145 and His41 upon binding of Z369936976 (×0397, magenta) compared to Z1129283193 (×0197, grey).

The experiment revealed one notable conformational variation, which was exploited by one fragment only (Z369936976 (×0397); Fig. 4): a change in the sidechains of the key catalytic residues His41, Cys145 alters the size and shape of subsite S1′ and thus the link to subsite S1. This allows the fragment to bind, uniquely, to both S1 and S1′. In S1, the isoxazole nitrogen hydrogen-bonds to His163, an interaction that features in several other hits; and in S1′, the cyclopropyl group occupies the region sampled by the covalent fragments. Notably, the N-methyl group offers a vector to access the S2 and S3 subsites.

It is established that the biological unit for similar viral proteases such as the SARS-CoV-1 protease is a dimer^38^, and that mutations at the dimer interface can disrupt proteases activity^39,40^ even at long range^41^. Thus, compounds that interfere with dimerization might serve as quasi-allosteric inhibitors of protease activity. In this study three compounds bound at accessible sites of the dimer interface, that conceivably could be exploited to design compounds to disrupt the M^pro^ dimer.

**Fig. 5:**
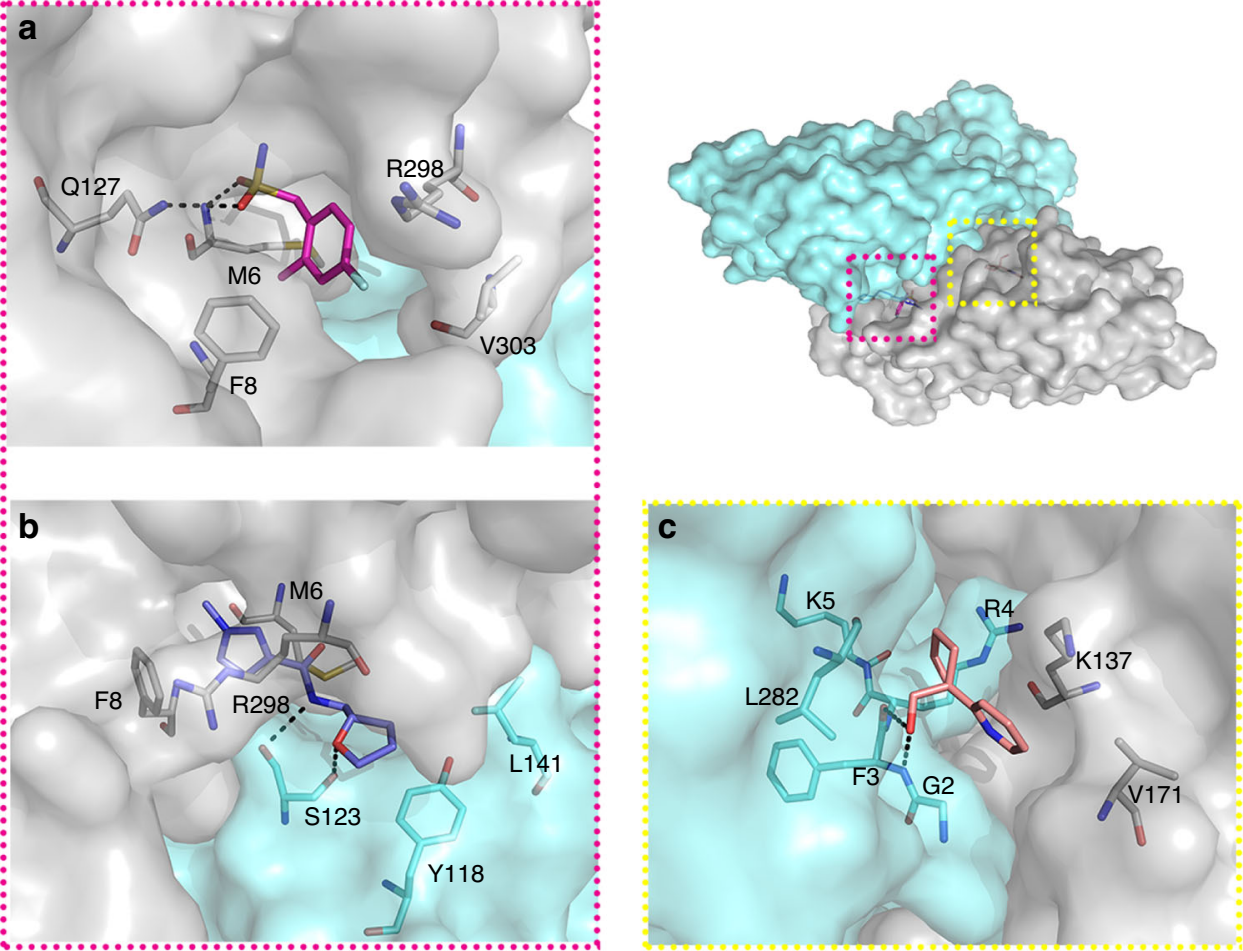
Fragments at dimer interface indicate opportunities for allosteric modulation. The overview shows the surface of the M^pro^ dimer, the protomers in grey and cyan. Fragments and surrounding residues are shown as sticks and hydrogen bonds in dashed black lines. **a** Z1849009686 (×1086). **b** Z264347221 (×1187). **c** POB0073 (×0887).

Fragment Z1849009686 (×1086; Fig. 5a) binds in a hydrophobic pocket formed by the sidechains of Met6, Phe8, Arg298 and Val303. It also mediates two hydrogen bonds to the sidechain of Gln127 and the backbone of Met6. Its binding site is <7 Å away from Ser139, whose mutation to alanine in SARS-CoV-1 protease reduced both dimerization and protease activity by about 50%^39,42^. Z264347221 (×1187, Fig. 5b) binds similarly in a hydrophobic pocket made by Met6, Phe8 and Arg298 in one of the protomers, extending across the dimer interface to interact with Ser123, Tyr118 and Leu141 of the second protomer, including hydrogen bonds with the sidechain and backbone of Ser123. Finally, POB0073 (x0887; York 3D library; Fig. 5c), binds only 4 Å from Gly2 at the dimer interface and is encased between Lys137 and Val171 of one protomer and Gly2, Arg4, Phe3, Lys5 and Leu282 of the second, including two hydrogen bonds with the backbone of Phe3.

### Covalent fragment hits reveal several tractable series

The screen further yielded 48 structures of fragments covalently bound to the nucleophilic active site Cys145, and substrate subsite S1´. The majority (44) fall into series explored in the mass-spectrometry experiment and the remainder came from other libraries.

**Fig. 6:**
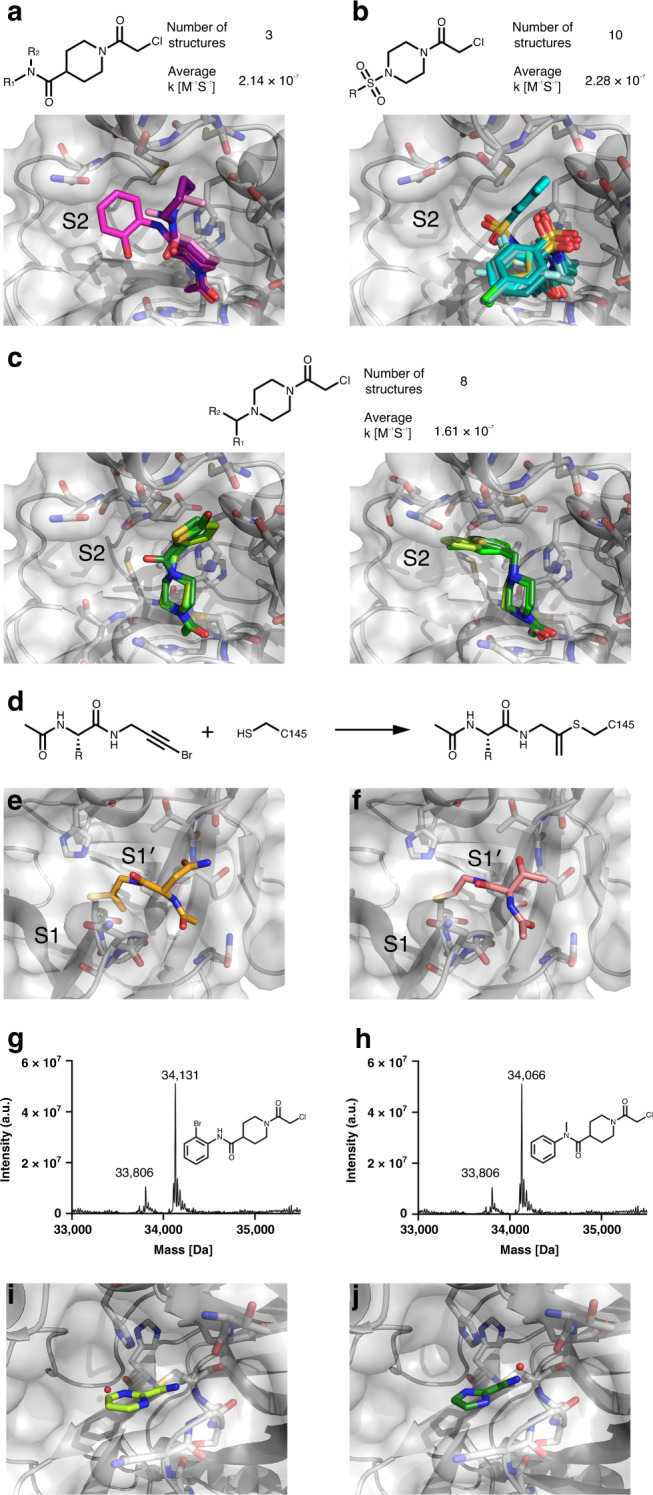
Covalent fragments are anchored at Cys145 and sample different regions of the orthosteric M^pro^ binding pocket. **a** Fragments containing *N*-chloroacetyl piperidinyl-4-carboxamide motif. **b** Fragments containing *N*-chloroacetyl-*N*´-sulfonamido-piperazine motif. **c** Fragments containing *N*-chloroacetyl-*N*´-carboxamido- and *N*-chloroacetyl-*N*´-heterobenzyl-piperazine in two binding modes. The second order kinetic constants refer to the intrinsic thiol reactivity of these fragment hits as previously measured^29^. **d** Reaction schema of the unexpected covalent modification to Cys145 by PepLites hits. **e** Threonine PepLite (NCL-00025058 (x0978)) bound covalently to active site cysteine. **f** Asparagine PepLite (NCL-00025412 (x0981)) bound to active site cysteine. Labelling of M^pro^ by 2nd generation compounds proven by intact protein LC-MS: **g** Labelling by PG-COV-35; **h** Labelling by PG-COV-34. Covalently bound cyclic electrophiles: **i** Cov_HetLib 030 (×2097) and **j** Cov_HetLib 053 (×2119).

In all structures with bound electrophiles, the *N*-chloroacetyl carbonyl oxygen atom forms either two or three hydrogen bonds with the backbone amide hydrogens of Gly143, Ser144 or Cys145 (Fig. 6a–c). All three compounds containing the *N*-chloroacetyl piperidinyl-4-carboxamide motif (Fig. 6a) adopt a similar binding mode pointing towards the S2 pocket, and one (PCM-0102389, ×1358) is able to form an additional hydrogen bond with the side chain of Asn142.

Compounds with the *N*-chloroacetyl-*N*’-sulfonamido-piperazine motif (Fig. 6b) adopt a bent shape, pointing towards the S2 pocket where appropriate space-filling substituents are attached to the phenyl moiety (PCM-0102353 (×1336) and PCM-0102395 (×0774)); otherwise, they point towards the solvent. Most of the latter 8 structures feature a halophenyl moiety which resides closely to Asn142, hinting at weak halogen-mediated interactions^43^.

Eight compounds with a *N*-chloroacetyl-*N*´-carboxamido- and *N*-chloroacetyl-*N*´-heterobenzyl-piperazine motif crystallized in one binding mode with respect to the piperazinyl moiety (Fig. 6c) (with one exception, PCM-0102287 (×0830)). Two structures (PCM-0102277 (×1334), PCM-0102169 (×1385)) with a 5-halothiophen-2-ylmethylene moiety exploit lipophilic parts of S2, which is also recapitulated by the thiophenyl moiety in an analogous carboxamide (PCM-0102306 (x1412)). The other five structures point mainly to S2, offering an accessible growth vector towards the nearby S3 pocket.

A series of compounds containing a N-chloroacetyl piperidinyl-4-carboxamide motif showed promising binding modes. To follow-up on these compounds, we performed rapid second-generation compound synthesis. Derivatives of this chemotype were accessible in milligram-scale by the reaction of N-chloroacetyl piperidine-4-carbonyl chloride with various in-house amines, preferably carrying a chromophore to ease purification. These new compounds were tested by intact protein mass-spectrometry to assess protein labelling (5 μM compound; 1.5 h incubation, RT; Supplementary Data 3). Amides derived from non-polar amines mostly outcompeted their polar counterparts, hinting at a targetable lipophilic sub-region in this direction. The two amides with the highest labelling PG-COV-35 and PG-COV-34 (Fig. 6g, h) highlight the potential for further synthetic derivatization by amide N-alkylation or cross-coupling, respectively.

The screen revealed unexpected covalent warheads from the series of 3-bromoprop-2-yn-1-yl amides of N-acylamino acids. Colloquially termed PepLites, this library was developed to map non-covalent interactions of amino acid sidechains in protein-protein interaction hotspots, with the acetylene bromine intended, as for FragLites^44,45^, as a detection tag by anomalous dispersion in X-ray crystallography. However, bromoalkynes can also act as covalent traps for activated cysteine thiols^46^ (Fig. 6d).

Two PepLites, containing threonine (NCL-00025058 (×0978)) and asparagine (NCL00025412 (×0981)) bound covalently to the active site cysteine (Cys145), forming a thioenolether via C-2 addition with loss of bromine (Fig. 6e, f). The covalent linkage was unexpected and evidently the result of significant non-covalent interactions, specific to these two PepLites, that position the electrophile group for nucleophilic attack. We note the side-chains make hydrogen-bonding interactions with various backbone NH and O atoms of Thr26 and Thr24; in the case of threonine, it was the minor 2R,3R diastereomer (corresponding to D-allothreonine) that bound. The only other PepLite observed (tyrosine, NCL-00024905 (×0967)) bound non-covalently to a different subsite.

The highlighted structure-activity relationships are important for further optimisation. Bromoalkynes have intrinsic thiol reactivity that is lower than that of established acrylamide-based covalent inhibitors^46^, which is in general desirable. The geometry of the alkyne and its binding mode also suggest that it could be replaced by reversible covalent groups such as nitriles, which would be guided by the same non-covalent interactions but are better established as cysteine protease inhibitors.

Two covalent hits (2-cyanopyrimidine (Cov_HetLib 030 (×2097)) and 2-cyanoimidazole (Cov_HetLib 053 (×2119) came from a library of small heterocyclic electrophiles^47^. These are essentially covalent MiniFrags^48^, comprising five and six-membered nitrogen-containing heterocycles with electron-withdrawing character that activates small electrophilic substituents (halogens, ethylyl, vinyl and nitrile groups).

Both hits bound to Cys145 through an imine (Fig. 6i, j), positioned by a local hydrogen bond network involving imine and heterocyclic N atoms. One of these free amines provides an immediate growth vector towards the catalytic pocket. The compounds have reasonable stability in water^49^ and limited reactivity against GSH (*t*_1/2_ = 2.2 and 52.3 h, respectively), well above suggested reactivity limits^50^. They are also inactive against various covalent targets (HDAC8, MAO-A, MAO-B, MurA) and benchmark proteins.

## Discussion

The data presented herein provide many clear routes to developing potent inhibitors of M^pro^ from SARS-CoV-2. The bound fragments comprehensively sample all subsites of the active site, revealing diverse expansion vectors, and the electrophiles provide extensive data, systematic as well as serendipitous, for designing covalent compounds.

It is widely accepted that new small-molecule drugs cannot be developed fast enough to help against COVID-19. Nevertheless, as the pandemic threatens to remain a long-term problem and vaccine candidates do not promise complete and lasting protection, antiviral molecules will remain an important line of defence. Such compounds will also be needed to fight future pandemics^10^. Our data will accelerate such efforts: therapeutically, through the design of new molecules and to inform ongoing efforts at repurposing existing drugs; and for research, through the development of probe molecules^51^ to understand viral biology. One example is the observation that fragment Z1220452176 (×0104) is a close analogue of melatonin, although in this case, it is unlikely that melatonin mediates direct antiviral activity through inhibition of M^pro^, given its low molecular weight; nevertheless, melatonin is currently in clinical trials to assess its immune-regulatory effects on COVID19 (Clinicaltrials.gov identifier NCT04353128).

In line with the urgency, results were made available online immediately for download. In addition, since exploring 3D data requires specialised tools^52,53^, hits were made accessible on the Fragalysis webtool (https://fragalysis.diamond.ac.uk) that allows easy exploration of the hits in interactive 3D.

All released models were stringently assessed for reliability. On the one hand, the whole data analysis process necessarily relied heavily on automation that, since its initial testing, has been extensively validated on over 100 experiments at the XChem facility, indicating the processes are robust in generating high-quality atomic models. On the other hand, the final selection of models was by subjective evaluation of the fit of each atomic model to electron density. All models were therefore reviewed by multiple authors prior to release, and a subjective confidence assigned to each (Supplementary Data 2). The evidence used was the unbiased event density generated by the PanDDA method^37^, which uses multi-dataset averaging to extract signal from electron density that would historically have been considered too noisy to be convincingly interpretable^54^; accordingly, even ligands with low occupancy (<40%) could be confidently assessed (Supplementary Note 1). Likewise this means that poor diffraction is a common occurrence due to the crystal handling steps required for soaking of fragments. However, for each dataset, the dominant source of noise is low occupancy and not phase bias, since crystals and thus datasets are only subtly different (Supplementary Note 1).

**Fig. 7:**
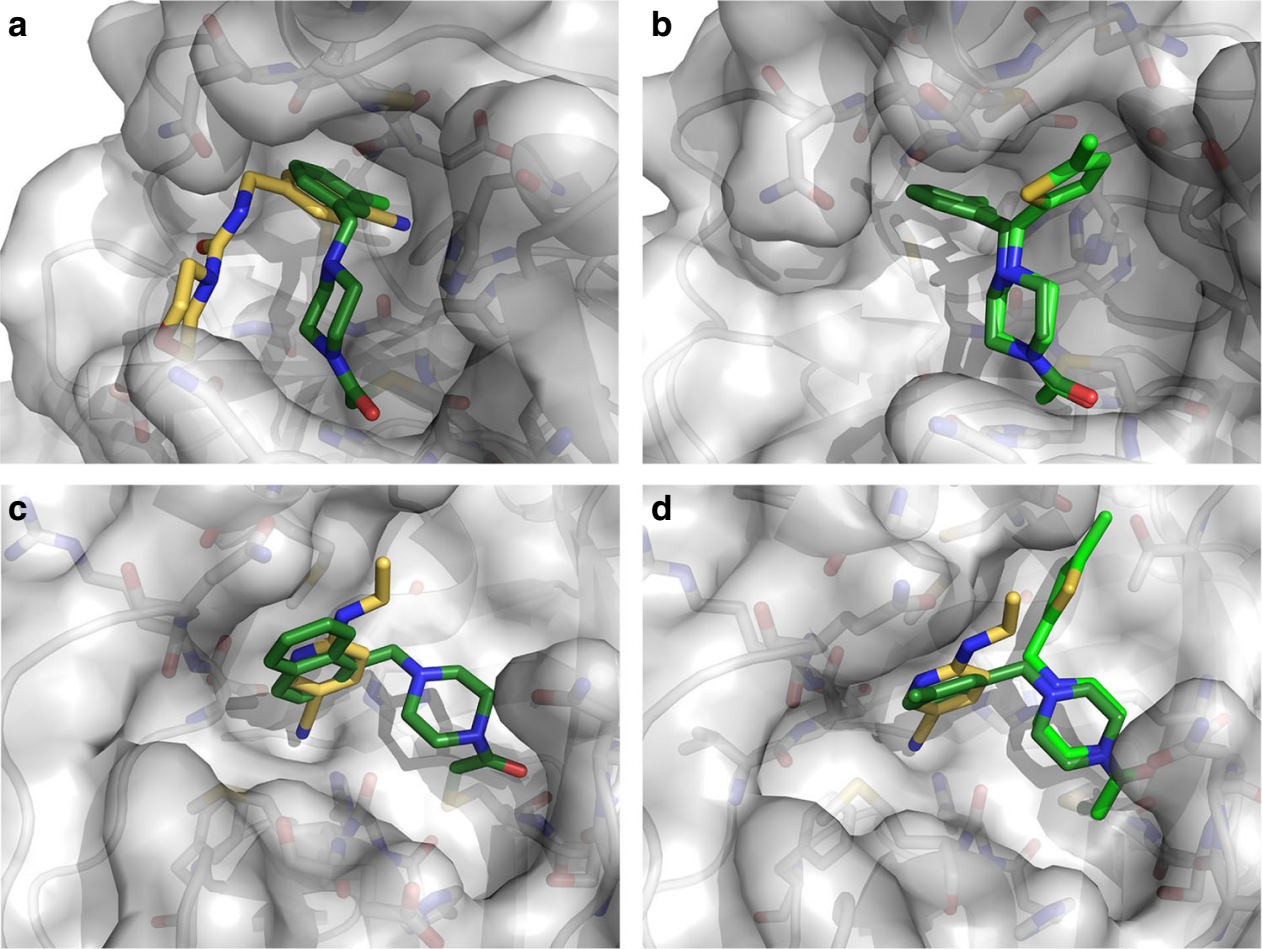
Fragment merging opportunities can be directly inferred from many hits. Covalently bound fragments are in green shades, and non-covalent fragments in yellow. **a** Overlay of Z509756472/×1249 and PCM-0102269/×0770. **b** Overlay of PCM-0102277/x1334 and PCM-0102269/×0770. **c** Overlay of PCM-0102287/×0830 and Z219104216/×0305. **d** Overlay of PCM-0102340/×0692, PCM-0102277/×1334 and Z219104216/×0305.

We have previously demonstrated the benefits of merging covalent and non-covalent fragments to make dramatic improvements in potency^29^. Our dataset offers numerous opportunities and some conservative examples are shown in Fig. 7. These can be expected to result in potent M^pro^ binders and compound synthesis is ongoing.

Collectively, the covalent hits provide rational routes to inhibitors of low reactivity and high selectivity. Rationally designed covalent drugs are gaining traction, with many recent FDA approvals^55,56^. Their design is based on very potent non-covalent binding, that allows precise orientation of a low reactivity electrophile, so that formation of the covalent bond is reliant on binding site specificity, with minimal off-target effects^57–59^. For fear of over-reactivity, covalent inhibitors are expunged from high-throughput screening libraries and are typically considered as PAINS compounds^60–62^. The challenge of tuning reactivity, and the danger of reactivity-based artefacts, are considered to be particularly marked for highly reactive nucleophiles such as the catalytic cysteine of many proteases. This is evidenced by the very high hit-rate we saw in our preliminary screen of electrophiles in which more than 150 fragments labelled M^pro^ by >50%. Robust characterization of the fragments’ reactivity^29^, and continuous evaluation of general thiol reactivity in the selection of lead series and during hit-to-lead optimization can address this challenge. We note here that most of the electrophiles that we screened (chloroacetamides and acrylamides) form irreversible adducts with the target cysteine, whereas many protease inhibitors contain aldehydes, nitriles and α-ketoamides, that can form reversible covalent bonds.

The scale of this experiment, particularly the diversity of libraries and density of results, likely sets a new benchmark for ensuring a crystal-based fragment screen accelerates progression of hits. Even cursory inspection of the fragment structures indicates a very large “merge space”, i.e., the collection of compounds that can be designed directly from spatial juxtapositions of fragments. Such merges, which can be made to populate all four subsites, might achieve potency synergistically, because the observed interactions can be assumed to be in near-optimal configurations, given how few there are per fragment. A thorough exploration of merge space might be best achieved formulaically, using computational workflows that additionally filter undesirable molecular properties, assess synthetic tractability and predict binding affinity. However, such integrated approaches are not currently available in the public domain. We hope this dataset will help spur their development and testing.

Another promising effort to explore the potential of this premise is the COVID Moonshot project (https://covid.postera.ai/covid), where a selection of merges will be experimentally tested, with data promptly made public. We trust that this resource will enable the development of many new tools, approaches and ultimately viable treatment candidates for COVID19.

## Methods

### Protein expression

The expression vector was constructed with a codon-optimised gene fragment, synthesized by Integrated DNA Technologies, which included in-fusion compatible ends for direct insertion into BamHI-XhoI digested pGEX-6P-1 (Supplementary Table 2). The resulting plasmid yields native N- and C-termini upon 3C protease treatment during the purification. Multiple transformant colonies were used to inoculate a starter culture supplemented with 100 µg/ml Carbenicillin. The culture was then grown to log phase for ~8 h. Ten millilitres of the starter culture was used to inoculate one litre of auto induction medium supplemented with 10 ml of glycerol and 100 µg/ml carbenicillin. The cultures were grown at 37 °C, 200 rpm for 5 h then switched to 18 °C, 200 rpm for 10 h. The cells were harvested by centrifugation and stored at −80 °C

### Protein purification

Cells were resuspended in 50 mM Tris pH 8, 300 mM NaCl, 10 mM Imidazole, 0.03 μg/ml Benzonase. The cells were disrupted on a high-pressure homogeniser (3 passes, 30 kpsi, 4 °C). The lysate was clarified by centrifugation at 50,000 × *g*. The supernatant was then applied to a Nickel-NTA gravity column and washed and eluted with 50 mM Tris pH 8, 300 mM NaCl, and 25–500 mM imidazole pH 8. N-terminal His-tagged HRV 3 C Protease was then added to the eluted protein at 1:10 w/w ratio. The mixture was then dialysed overnight at 4 °C against 50 mM Tris pH 8, 300 mM NaCl, 1 mM TCEP. The following day, the HRV 3 C protease and other impurities were removed from the cleaved target protein by reverse Nickel-NTA. The relevant fractions were concentrated and applied to an S200 16/60 gel filtration column equilibrated in 20 mM Hepes pH 7.5, 50 mM NaCl buffer. The protein was concentrated to 30 mg/ml using a 10 kDa MWCO centrifugal filter device.

### Crystallisation and structure determination

Protein was thawed and diluted to 5 mg/ml using 20 mM Hepes pH 7.5, 50 mM NaCl. The sample was centrifuged at 100,000 × *g* for 15 min. Initial hits were found in well F2 of the Proplex crystallisation screen, 0.2 M LiCl, 0.1 M Tris pH 8, 20% PEG 8 K. These crystals were used to prepare a seed stock by crushing the proteins with a pipette tip, suspending in reservoir solution and vortexing for 60 s in the reservoir solution with ~10 glass beads (1.0 mm diameter, BioSpec products). Adding DMSO to the protein solution to a concentration of 5% and performing microseed matrix screening, many new crystallisation hits were discovered in commercial crystallisation screens. Following optimisation, the final crystallisation condition was 11% PEG 4 K, 6% DMSO, 0.1 M MES pH 6.7 with a seed stock dilution of 1/640. The seeds were prepared from crystals grown in the final crystallisation condition. The drop ratios were 0.15 µl protein, 0.3 µl reservoir solution, 0.05 µl seed stock. Crystals were grown using the sitting drop vapour diffusion method at 20 °C and appeared within 24 h.

Initial diffraction data were collected on beamline I04 at Diamond Light Source on a crystal grown in 0.1 M MES pH 6.5, 5% PEG6K, cryoprotected using 30% PEG400. Data were processed using Dials^63^ via Xia2^64^. The dataset was phased with the SARS-CoV-2 M^pro^ in complex with the N3 inhibitor crystal structure (PDB:6LU7 [10.2210/pdb6lu7/pdb]) using Molrep in CCP4i2. Further datasets were collected on I04-1 at Diamond Light Source on crystals grown using the 0.1 M MES pH 6.5, 15% PEG4K, 5% DMSO condition. To create a high-resolution dataset, datasets from 7 crystals were scaled and merged using Aimless^65^. Crystal structures were manually rebuilt in Coot^66^ and refined using Refmac^67^ and Buster^68^. This structure is deposited in the PDB under ID 6YB7 [10.2210/pdb6yb7/pdb].

### Electrophile fragment LC/MS screen

2 µM M^pro^ was incubated in 50 mM Tris pH 8 300 mM NaCl for 1.5 h at 25 °C. For initial electrophile fragment library screen, 30 µl protein with pools of 4–5 electrophile fragments, 7.5 nL each from 20 mM DMSO stocks and for other runs 50 µl protein with 0.5 µl compounds from 0.5 mM DMSO stocks. The reaction was quenched by adding formic acid to 0.4% final concentration. The LC/MS runs were performed on a Waters ACUITY UPLC class H instrument, in positive ion mode using electrospray ionization. UPLC separation used a C4 column (300 Å, 1.7 μm, 21 mm × 100 mm). The column was held at 40 °C and the autosampler at 10 °C. Mobile solution A was 0.1% formic acid in water, and mobile phase B was 0.1% formic acid in acetonitrile. The run flow was 0.4 mL/min with gradient 20% B for 4 min, increasing linearly to 60% B for 2 min, holding at 60% B for 0.5 min, changing to 0% B in 0.5 min, and holding at 0% for 1 min. The mass data were collected on a Waters SQD2 detector with an *m/z* range of 2 − 3071.98 at a range of 1000–2000 *m/z*. Raw data were processed using openLYNX and deconvoluted using MaxEnt. For each well, deconvoluted peaks were searched to match either the unlabelled protein, or labelled protein with one or two of the compounds in the well. Labelling percentage for a compound was determined as the % of a specific compound adduct, divided by the overall detected protein species. Peaks whose mass could not be assigned were discarded from the overall labelling calculation. Wells are regarded as “bad wells” if their spectra appeared to be of a degraded protein (low intensity and deformed peak shape) or if after deconvolution there were no clear peaks (high noise levels). No labelling was assigned for bad wells.

### Fragment screening

Fragments were soaked into crystals by adding dissolved compound directly to the crystallisation drops. The following libraries were screened: the DSipoised library (Enamine), a version of the poised library^25^; a version of the MiniFrags library^48^ assembled in-house; the FragLites library^45^; a library of shape-diverse 3D fragments (“York3D”)^69^; heterocyclic electrophiles^47^; and the SpotFinder library. All fragments were in 100% DMSO at varying stock concentrations, detailed at https://www.diamond.ac.uk/Instruments/Mx/Fragment-Screening/Fragment-Libraries.html). In brief, 55 nl of fragment stock solutions in DMSO (DSI-poised, FragLite, PepLites, York 3D, Covalent Heterocylces and SpotFinder all at 500 mM, MiniFrags at 1 M and Cysteine covalent library at 20 mM) were transferred directly to 500 nl crystallisation drops using an ECHO liquid handler giving a final compound concentration of 2–100 mM and DMSO concentration of 10%. Drops were incubated at room temperature for ~1 h prior to mounting and flash cooling in liquid nitrogen without the addition of further cryoprotectant.

Electrophile fragments identified by mass spectrometry were soaked by the same procedure as the other libraries, but in addition, they were also co-crystallised in the same crystallisation condition as for the apo structure. The protein was incubated with 10–20-fold excess compound (molar ratio) for ~1 h prior to the addition of the seeds and reservoir solution (following Resnick et al.^29^).

Data were collected at the beamline I04-1 at 100 K and processed with the fully automated pipelines at Diamond^63,70,71^, which variously combine XDS^72^, xia2^64^, autoPROC^73^ and DIALS^63^, and select resolution limits algorithmically; no manual curation of processing parameters was applied. Further analysis was performed through XChemExplorer^27^: for each dataset, the version of processed data was selected by the default XChemExplorer score, and electron density maps were generated with Dimple^74^. Ligand-binding events were identified using PanDDA^37^ (both the released version 0.2 and a pre-release development version (https://github.com/ConorFWild/pandda)), and ligands were modelled into PanDDA-calculated event maps using Coot^66^. Restraints were calculated with ACEDRG or GRADE^75,76^, structures were refined with Refmac^67^ and Buster^68^, and models and quality annotations cross-reviewed. Further elaboration of the PanDDA analysis is provided in the Supplementary Note 1.

Coordinates, structure factors and PanDDA event maps for all data sets are deposited in the Protein Data Bank under group deposition ID G_1002135, G_1002151, G_1002152, G_1002153, G_1002156 and G_1002157. Data collection and refinement statistics are summarised in Supplementary Data 4. The ground-state structure and all corresponding datasets are deposited under PDB ID 5R8T [10.2210/pdb5r8t/pdb].

### Synthesis of *N*-chloroacetyl-piperidine-4-carboxamides

*N*-chloroacetyl piperidine-4-carbonyl chloride was prepared as a stock solution in dry DCM under an atmosphere of N_2_. In brief, deprotecting *N*-Boc isonepecotic acid in 50% TFA in DCM (v/v) at RT for 2 h yielded the corresponding TFA salt after evaporation of all volatiles. The crude TFA salt was then re-dissolved in DCM, treated with Et_3_N (2 equiv.), followed by the addition of chloroacetic anhydride (1 equiv.). The reaction mixture was stirred overnight at RT, washed with water, the organic phase dried over MgSO_4_, filtered, and all volatiles removed by rotary evaporation. The crude N-chloroacetyl piperidine-4-carboxylic acid was refluxed in excess neat SOCl_2_ (gas evolution and a colour change to red occurs) for 1 h, followed by removal of excess SOCl_2_ in vacuum into a liquid nitrogen-cooled trap. The remaining residue was dried by rotary evaporation, placed under an atmosphere of nitrogen and dissolved in dry DCM to give a stock solution of ~0.489 M (based on theoretical yield over three steps), which was immediately used.

The corresponding amides were prepared by the addition of the acid chloride (1 equiv.) as a DCM solution to the pertinent amines (1 equiv.) in presence of pyridine (1 equiv.) in DCM. Heterogeneous reaction mixtures were treated with a minimal amount of dry DMF to achieve full solubility. After stirring the reaction mixtures overnight, the solvents were removed in by rotary evaporation, re-dissolved in 50% aq. MeCN (and a minimal amount of DMSO to achieve higher solubility), followed by purification by (semi-)preparative RP-HPLC in mass-directed automatic mode or manually.

### Synthesis of PepLites

HATU (1.5 equiv.), DIPEA (3.0 equiv.) and the acid starting material (1.5 equiv.) were dissolved in DMF (3–6 mL) and stirred together at room temperature for 10 min. 3-Bromoprop-2-yn-1-amine hydrochloride was added and the reaction mixture was stirred at 40 °C overnight. The reaction mixture was allowed to cool to room temperature, diluted with EtOAc or DCM and washed with saturated aqueous sodium bicarbonate solution, brine and water. The organic layer was dried over MgSO_4_, filtered and evaporated to afford crude product. The crude product was then purified by either normal or reverse-phase chromatography.

### tert-Butyl (3-bromoprop-2-yn-1-yl)carbamate

A solution of KOH (2.7 g, 48 mmol) in water (15 mL) was added dropwise to a solution of *N*-bocpropargylamine (3.0 g, 19 mmol) in MeOH (45 mL) at 0 °C under nitrogen. The resulting solution was stirred at 0 °C for 10 min then bromine (1.1 mL, 21 mmol) was added dropwise. The reaction mixture was allowed to warm to room temperature and was stirred at room temperature for 24 h. The reaction mixture was diluted with water and extracted with diethyl ether. The organic extracts were combined, dried over MgSO_4_ and evaporated to afford crude product. The crude product was purified by flash silica chromatography, elution gradient 0–10% EtOAc in petroleum ether. Pure fractions were evaporated to dryness to afford *tert*-Butyl (3-bromoprop-2-yn-1-yl)carbamate (3.5 g, 79%) as a white solid. *R*_f_ = 0.34 (10% EtOAC in petroleum ether); m.p. 108–110 °C; IR ν_max_ (cm^−1^) 3345, 2982, 2219, 2121, 2082; ^1^H NMR (500 MHz, DMSO-*d*6) δ 1.39 (s), 3.76 (d, *J* = 5.9 Hz), 7.30 (d, *J* = 6.1 Hz); ^13^C NMR (126 MHz, DMSO-*d*_6_) δ 28.63, 30.89, 43.44, 78.46, 78.81, 155.69; LCMS *m/z* ES^+^ [M-Boc+H]^+^ 133.9; HRMS calcd for C_8_H_12_^79^BrNO_2_ 255.9949 [M(Br)+Na]^+^ found 256.0209.

### 3-Bromoprop-2-yn-1-amine hydroch**l**oride

*tert*-Butyl (3-bromoprop-2-yn-1-yl)carbamate (1.1 g, 4.7 mmol) was dissolved in 4 M HCl in dioxane (30 mL). The reaction mixture was stirred at room temperature for 2 h then evaporated to dryness to afford 3-bromoprop-2-yn-1-amine hydrochloride (0.79 g, 99%) as a yellow solid. m.p. 169 °C; IR ν_max_ (cm^−1^) 2856, 2629, 2226, 2121, 2074; ^1^H NMR (500 MHz, DMSO-*d*_6_) δ 3.78 (s, 2H), 8.48 (s, 3H); ^13^C NMR (126 MHz, DMSO-*d*_6_) δ 29.69, 49.38, 73.90; LCMS m/z ES^+^ [M + H]^+^ 134.0; HRMS calcd for C_3_H_5_^79^BrN 1339605 [M(Br)+H]^+^ found 133.9598.

### (2S,3R)-2-Acetamido-*N*-(3-bromoprop-2-yn-1-yl)-3-(*tert*-butoxy)butanamide

(2S, 3S)-2-Acetamido-N-(3-bromoprop-2-yn-1-yl)-3-(tert-butoxy)butanamide was synthesized according to General procedure A using (2*S*,3*R*)-2-acetamido-3-(*tert*-butoxu)butanoic acid (0.41 g, 1.9 mmol). The crude product was purified by flash silica chromatography, elution gradient 0–10% MeOH in DCM. Pure fractions were evaporated to dryness to afford (2*S*, 3*S*)-2-acetamido-*N*-(3-bromoprop-2-yn-1-yl)-3-(*tert*-butoxy)butanamide (0.20 g, 42%) as a white solid. R_f_ = 0.46 (10% MeOH in DCM); mp: 180–183 °C; IR ν_max_ (cm^−1^) 3271, 3078, 2969, 2935, 2222, 2113; ^1^H NMR (500 MHz, Methanol-*d*_4_) δ 1.16 (d, *J* = 6.2, 5.0 Hz), 1.21 (s, *J* = 3.9 Hz, 9H), 2.01 (s, 3H), 3.91–4.09 (m, 3H), 4.32 (d, *J* = 7.5 Hz, 1H); ^13^C NMR (126 MHz, Methanol-*d*_4_) δ 18.61, 21.15, 27.27, 28.90, 41.92, 58.81, 67.21, 74.16, 75.57, 171.19, 171.92; LCMS m/z ES + [M + H] + 333.2; HRMS calcd for C_13_H_21_^79^BrN_2_O_3_ 333.2260 [M(Br)+H]^+^ found 333.0808.

### (2S,3R)-2-Acetamido-N-(3-bromoprop-2-yn-1-yl)-3-hydroxybutanamide (threonine PepLite)

(2*S*,3*S*)-2-Acetamido-*N*-(3-bromoprop-2-yn-1-yl)-3-(*tert*-butoxy)butanamide (80 mg, 0.24 mmol) was dissolved in anhydrous DCM (20 mL) and TFA (10 mL) and 0 °C under nitrogen. The reaction mixture was allowed to warm to room temperature and was stirred at room temperature for 3 h then evaporated to dryness to afford crude product. The crude product was purified by flash silica chromatography, elution gradient 0–15% MeOH in DCM. Pure fractions were evaporated to dryness to afford (2*S*,3*S*)-2-acetamido-*N*-(3-bromoprop-2-yn-1-yl)-3-hydroxybutanamide (38 mg, 57%, 93% de) as a white solid. R_f_ = 0.34 (10% MeOH in DCM); mp: 189–192 °C; IR ν_max_ (cm^−1^) 3280, 3085, 2973, 2924, 2225, 2115; ^1^H NMR (500 MHz, Methanol-*d*_4_) δ 1.21 (d, *J* = 6.4 Hz, 3H), 2.03 (s, 3H), 3.97 – 4.06 (m, 3H), 4.33 (d, *J* = 6.5 Hz, 1H); ^13^C NMR (126 MHz, Methanol-*d*_4_) δ 18.21, 21.13, 29.00, 41.79, 58.69, 67.11, 75.41, 170.88, 172.00; LCMS m/z ES^+^ [M + H]^+^ 277.1; HRMS calcd for C_9_H_13_^79^BrN_2_O_3_ 277.1180 [M(Br)+H]^+^ found 277.0182.

### (*S*)-2-Acetamido-*N*^1^-(3-bromoprop-2-yn-1-yl)succinimide (asparagine PepLite)

(*S*)-2-Acetamido-*N*^1^-(3-bromoprop-2-yn-1-yl)succinamide was synthesized according to General procedure A using (*s*)-2-acetamido-5-amino-5-oxobutanoic acid (155 mg, 0.89 mmol) and evaporating the reaction mixture to afford the crude product without aqueous work-up. The crude product was purified by flash silica chromatography, elution gradients 0–10% MeOH in DCM. Pure fractions were evaporated to dryness to afford (*S*)-2-acetamido-*N*^1^-(3-bromoprop-2-yn-1-yl)succinamide (50 mg, 30%) as a white solid. R_f_ = 0.18 (10% MeOH in DCM); mp: 173 °C (decomp); IR ν_max_ (cm^−1^) 3421, 3277, 3208, 3072, 2922, 2226, 2116; 1H NMR (500 MHz, Methanol-d4) δ 1.99 (s, 3H), 2.58–2.75 (m, 2H), 3.98 (d, *J* = 1.4 Hz, 2H), 4.71 (dd, *J* = 7.6, 5.7 Hz, 1H); 13 C NMR (126 MHz, Methanol-d4) δ 22.57, 30.61, 37.83, 43.13, 51.54, 76.84, 173.04, 173.28, 174.81; LCMS m/z ES^+^ [M + H]^+^ 290.2; HRMS calcd for C_9_H_12_^79^BrN_3_O_3_ 290.1170 [M(Br)+H]^+^ found 290.2265.

### Reporting summary

Further information on research design is available in the Nature Research Reporting Summary linked to this article.

## Supplementary information

Supplementary Information

Peer Review File

Description of Additional Supplementary Files

Supplementary Data 1

Supplementary Data 2

Supplementary Data 3

Supplementary Data 4

Reporting summary

## Data Availability

The coordinates and structure factors have been deposited in the Protein Data Bank. The accession codes are listed in Supplementary Data 2. Other data are available from the corresponding authors upon reasonable request.

## References

[1] Wu F (2020). A new coronavirus associated with human respiratory disease in China. Nature.

[2] Kucharski AJ, Russell TW, Diamond C (2020). Early dynamics of transmission and control of COVID-19: a mathematical modelling study (vol 20, pg 553, 2020). Lancet Infect. Dis..

[3] Zhu N (2020). A novel coronavirus from patients with pneumonia in China, 2019. N. Engl. J. Med.

[4] Dong ES, Du HR, Gardner L (2020). An interactive web-based dashboard to track COVID-19 in real time. Lancet Infect. Dis..

[5] Bermingham A (2012). Severe respiratory illness caused by a novel coronavirus, in a patient transferred to the United Kingdom from the Middle East, September 2012. Eurosurveillance.

[6] Kuiken T (2003). Newly discovered coronavirus as the primary cause of severe acute respiratory syndrome. Lancet.

[7] Zaki AM, van Boheemen S, Bestebroer TM, Osterhaus ADME, Fouchier RAM (2012). Isolation of a novel coronavirus from a man with pneumonia in Saudi Arabia. N. Engl. J. Med..

[8] Bredenbeek PJ (1990). The primary structure and expression of the 2nd open reading frame of the polymerase gene of the coronavirus Mhv-A59 - a Highly conserved polymerase is expressed by an efficient ribosomal frameshifting mechanism. Nucleic Acids Res..

[9] Thiel V (2003). Mechanisms and enzymes involved in SARS coronavirus genome expression. J. Gen. Virol..

[10] Hilgenfeld RFrom (2014). SARS to MERS: crystallographic studies on coronaviral proteases enable antiviral drug design. Febs J..

[11] Ghosh AK (2007). Structure-based design,synthesis, and biological evaluation of peptidomimetic SARS-CoV 3CLpro inhibitors. Bioorg. Med. Chem. Lett..

[12] Verschueren KHG (2008). A structural view of the inactivation of the SARS coronavirus main proteinase by benzotriazole esters. Chem. Biol..

[13] Yang HT (2005). Design of wide-spectrum inhibitors targeting coronavirus main proteases. PLoS Biol..

[14] Yang HT (2003). The crystal structures of severe acute respiratory syndrome virus main protease and its complex with an inhibitor. Proc. Natl Acad. Sci. USA.

[15] Anand K, Ziebuhr J, Wadhwani P, Mesters JR, Hilgenfeld R (2003). Coronavirus main proteinase (3CL(pro)) structure: basis for design of anti-SARS drugs. Science.

[16] Hegyi A, Ziebuhr J (2002). Conservation of substrate specificities among coronavirus main proteases. J. Gen. Virol..

[17] Stadler K (2003). SARS—Beginning to understand a new virus. Nat. Rev. Microbiol..

[18] Xue XY (2008). Structures of two coronavirus main proteases: implications for substrate binding and antiviral drug design. J. Virol..

[19] Zhang LL (2020). Crystal structure of SARS-CoV-2 main protease provides a basis for design of improved alpha-ketoamide inhibitors. Science.

[20] Zhang L (2020). alpha-Ketoamides as broad-spectrum inhibitors of coronavirus and enterovirus replication: structure-based design, synthesis, and activity assessment. J. Med. Chem..

[21] Severson WE (2007). Development and validation of a high-throughput screen for inhibitors of SARS CoV and its application in screening of a 100,000-compound library. J. Biomol. Screen.

[22] Thomas SE (2019). Structure-guided fragment-based drug discovery at the synchrotron: screening binding sites and correlations with hotspot mapping. Philos. T R Soc. A.

[23] Erlanson DA, Fesik SW, Hubbard RE, Jahnke W, Jhoti H (2016). Twenty years on: the impact of fragments on drug discovery. Nat. Rev. Drug Discov..

[24] Helliwell JR, Mitchell EP (2015). Synchrotron radiation macromolecular crystallography: science and spin-offs. IUCrJ.

[25] Cox OB (2016). A poised fragment library enables rapid synthetic expansion yielding the first reported inhibitors of PHIP(2), an atypical bromodomain. Chem. Sci..

[26] Collins PM (2017). Gentle, fast and effective crystal soaking by acoustic dispensing. Acta Crystallogr. Sect. D.-Struct. Biol..

[27] Krojer T (2017). The XChemExplorer graphical workflow tool for routine or large-scale protein-ligand structure determination. Acta Crystallogr. Sect. D.-Struct. Biol..

[28] Wright, N. D. et al. The low-cost, semi-automated shifter microscope stage transforms speed and robustness of manual protein crystal harvesting. Preprint at https://www.biorxiv.org/content/10.1101/2019.12.20.875674v1 (2019). 10.1107/S2059798320014114 PMC7787106 33404526

[29] Resnick E (2019). Rapid covalent-probe discovery by electrophile-fragment screening. J. Am. Chem. Soc..

[30] Shi JH, Song JX (2006). The catalysis of the SARS 3C-like protease is under extensive regulation by its extra domain. Febs J..

[31] Jin, Z. et al. Structure of M(pro) from SARS-CoV-2 and discovery of its inhibitors. *Nature* **582**, 289–293 (2020). 10.1038/s41586-020-2223-y 32272481

[32] Nonoo RH, Armstrong A, Mann DJ (2012). Kinetic template-guided tethering of fragments. ChemMedChem.

[33] Kathman SG, Xu Z, Statsyuk AV (2014). A fragment-based method to discover irreversible covalent inhibitors of cysteine proteases. J. Med. Chem..

[34] Kathman SG (2015). A small molecule that switches a ubiquitin ligase from a processive to a distributive enzymatic mechanism. J. Am. Chem. Soc..

[35] Johansson H (2019). Fragment-based covalent ligand screening enables rapid discovery of inhibitors for the RBR E3 ubiquitin ligase HOIP. J. Am. Chem. Soc..

[36] Backus KM (2016). Proteome-wide covalent ligand discovery in native biological systems. Nature.

[37] Pearce, N. M. et al. A multi-crystal method for extracting obscured crystallographic states from conventionally uninterpretable electron density. *Nat. Commun.* **8**, 15123 (2017). 10.1038/ncomms15123 PMC5413968 28436492

[38] Chou CY (2004). Quaternary structure of the severe acute respiratory syndrome (SARS) coronavirus main protease. Biochem.-Us.

[39] Chen S (2008). Residues on the dimer interface of SARS coronavirus 3C-like protease: dimer stability characterization and enzyme catalytic activity analysis. J. Biochem..

[40] Hsu WC (2005). Critical assessment of important regions in the subunit association and catalytic action of the severe acute respiratory syndrome coronavirus main protease. J. Biol. Chem..

[41] Barrila J, Bacha U, Freire E (2006). Long-range cooperative interactions modulate dimerization in SARS 3CL(pro). Biochemistry.

[42] Hu TC (2009). Two adjacent mutations on the dimer interface of SARS coronavirus 3C-like protease cause different conformational changes in crystal structure. Virology.

[43] Kuhn B, Gilberg E, Taylor R, Cole J, Korb O (2019). How significant are unusual protein-ligand interactions? Insights from database mining. J. Med. Chem..

[44] Bauman JD, Harrison JJEK, Arnold E (2016). Rapid experimental SAD phasing and hot-spot identification with halogenated fragments. Iucrj.

[45] Wood DJ (2019). FragLites-minimal, halogenated fragments displaying pharmacophore doublets. an efficient approach to druggability assessment and hit generation. J. Med. Chem..

[46] Mons E (2019). The alkyne moiety as a latent electrophile in irreversible covalent small molecule inhibitors of cathepsin K. J. Am. Chem. Soc..

[47] Keeley A, Abranyi-Balogh P, Keseru GM (2019). Design and characterization of a heterocyclic electrophilic fragment library for the discovery of cysteine-targeted covalent inhibitors. Medchemcomm.

[48] O’Reilly M (2019). Crystallographic screening using ultra-low-molecular-weight ligands to guide drug design. Drug Discov. Today.

[49] Keeley A (2018). Heterocyclic electrophiles as new MurA inhibitors. Arch. Pharm..

[50] Fuller N (2016). An improved model for fragment-based lead generation at AstraZeneca. Drug Discov. Today.

[51] Arrowsmith CH (2015). The promise and peril of chemical probes. Nat. Chem. Biol..

[52] Ferla MP, Pagnamenta AT, Damerell D, Taylor JC, Marsden BD (2020). MichelaNglo: sculpting protein views on web pages without coding. Bioinformatics.

[53] Lee WH (2011). Interactive JIMD articles using the iSee concept: turning a new page on structural biology data. J. Inherit. Metab. Dis..

[54] Pearce NM, Krojer T, von Delft F (2017). Proper modelling of ligand binding requires an ensemble of bound and unbound states. Acta Crystallogr. D. Struct. Biol..

[55] Singh J, Petter RC, Baillie TA, Whitty A (2011). The resurgence of covalent drugs. Nat. Rev. Drug Discov..

[56] Bauer RA (2015). Covalent inhibitors in drug discovery: from accidental discoveries to avoided liabilities and designed therapies. Drug Discov. Today.

[57] De Cesco S, Kurian J, Dufresne C, Mittermaier AK, Moitessier N (2017). Covalent inhibitors design and discovery. Eur. J. Med. Chem..

[58] Zhang T, Hatcher JM, Teng M, Gray NS, Kostic M (2019). Recent advances in selective and irreversible covalent ligand development and validation. Cell Chem. Biol..

[59] Baillie TA (2016). Targeted covalent inhibitors for drug design. Angew. Chem. Int Ed. Engl..

[60] Sirois S, Hatzakis G, Wei D, Du Q, Chou KC (2005). Assessment of chemical libraries for their druggability. Comput. Biol. Chem..

[61] Baell JB, Nissink JWM (2018). Seven year itch: pan-assay interference compounds (PAINS) in 2017-utility and limitations. ACS Chem. Biol..

[62] Baell JB, Holloway GA (2010). New substructure filters for removal of pan assay interference compounds (PAINS) from screening libraries and for their exclusion in bioassays. J. Med. Chem..

[63] Winter G (2018). DIALS: implementation and evaluation of a new integration package. Acta Crystallogr. Sect. D.-Struct. Biol..

[64] Winter G, Lobley CMC, Prince SM (2013). Decision making in xia2. Acta Crystallogr. D..

[65] Evans PR, Murshudov GN (2013). How good are my data and what is the resolution?. Acta Crystallogr. D..

[66] Emsley P, Lohkamp B, Scott WG, Cowtan K (2010). Features and development of Coot. Acta Crystallogr. D..

[67] Murshudov GN (2011). REFMAC5 for the refinement of macromolecular crystal structures. Acta Crystallogr. Sect. D.-Struct. Biol..

[68] Buster v. 2.10.13 (Cambridge, United Kingdom, 2017).

[69] Downes TD (2020). Design and synthesis of 56 shape-diverse 3D fragments. Chemistry.

[70] Winter G (2019). How best to use photons. Acta Crystallogr. D. Struct. Biol..

[71] Winter G, McAuley KE (2011). Automated data collection for macromolecular crystallography. Methods.

[72] Kabsch W (2010). Integration, scaling, space-group assignment and post-refinement. Acta Crystallogr. D..

[73] Vonrhein C (2011). Data processing and analysis with the autoPROC toolbox. Acta Crystallogr. Sect. D.-Struct. Biol..

[74] Keegan R, Wojdyr M, Winter G, Ashton A (2015). DIMPLE: a difference map pipeline for the rapid screening of crystals on the beamline. Acta Crystallogr. A.

[75] Long F (2017). AceDRG: a stereochemical description generator for ligands. Acta Crystallogr. Sect. D.-Struct. Biol..

[76] grade v. 1.2.19 (Global Phasing Ltd., Cambridge, United Kingdom, 2010).

