# Supplementary material for: Crystallographic and electrophilic fragment screening of the SARS-CoV-2 main protease
Source: Nat Commun. 2020 Oct 7;11:5047. doi: 10.1038/s41467-020-18709-w (PMC7542442; doi:10.1038/s41467-020-18709-w)
Supplement: Supplementary file 1 — Supplementary Information [file 41467_2020_18709_MOESM1_ESM.pdf]

Supplementary Note 1

PanDDA algorithm facilitates identification of weakly bound ligands

All datasets were analysed using the Pan Dataset Density Analysis (PanDDA) method[1]. The PanDDA algorithm takes advantage of the large number of datasets collected during a fragment campaign to detect partial-occupancy ligands that are not visible in normal crystallographic maps (Supplementary Fig. 1). PanDDA uses a statistical analysis to identify bound ligands, and then generates an "event map" for the bound state of the crystal. An event map approximates what would be observed if the ligand was bound at full occupancy and is generated by subtracting the unbound fraction of the crystal from the partial-occupancy dataset. Ligand occupancy is initially estimated by PanDDA using the Background Density Correction factor (BDC) that maximises the density signal[1], but the final occupancy comes from refinement.

While most of the fragments are also obvious in conventional maps, several ligands may appear unjustifiably modelled when only the corresponding 2mFo–DFc is inspected. Therefore, PanDDA event maps are included in the MMCIF file that can be downloaded from the PDB website. Additionally, each deposition of models analysed with PanDDA is accompanied by a separate deposition of the ground-state model which also contains structure factors of all the collected datasets and which can be used to reproduce the analysis. Finally, PanDDA models were refined as composite models consisting of the ligand-bound and confounding ground-state[2], yet deposited to the PDB as only the bound-state fraction, since the composite models are often complex and difficult to interpret; as a result, some quality indicators recalculated by the PDB (in particular R/Rfree) are slightly elevated R/Rfree values in some cases. The electron density evidence was made available in a generally accessible format online, at https://www.diamond.ac.uk/covid-19/for-scientists/Main-protease-structure-and-XChem/Electron-density-evidence.html.

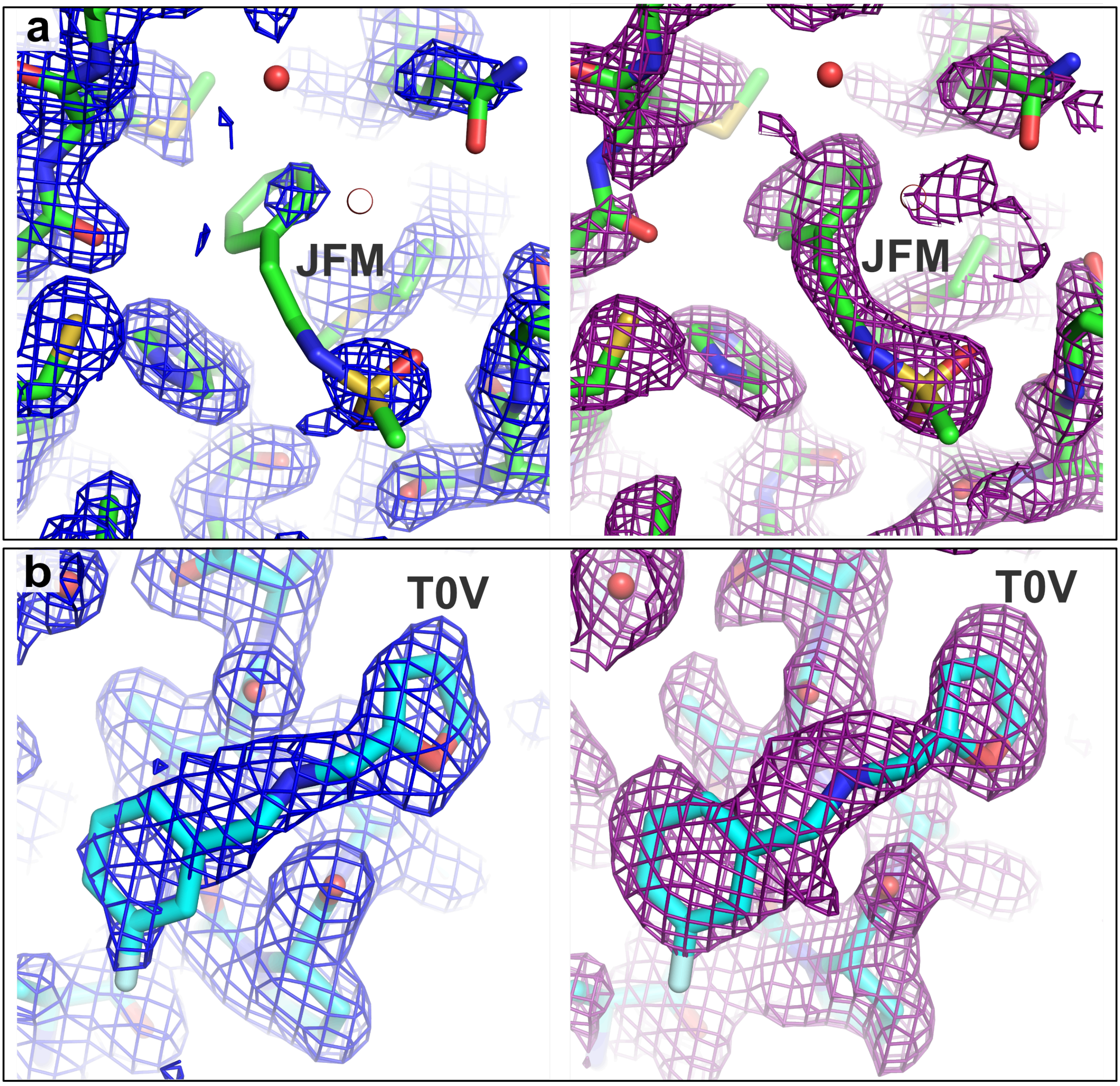

Supplementary Fig. 1. PanDDA event maps enable discovery of weakly bound fragments. The figure shows two examples of a weakly and a strongly bound ligand. Conventional 2mFo–DFc maps contoured at 1 σ are shown on the left side (blue), whereas the corresponding PanDDA event map is shown on the right side (purple). **a** Example of a weakly bound ligand (JFM) and how the PanDDA event map served as evidence for the deposited structure (PDB ID 5R7Y). **b** Example of a ligand (T0V) that is clearly visible in a conventional crystallographic map (PDB ID 5RE8)

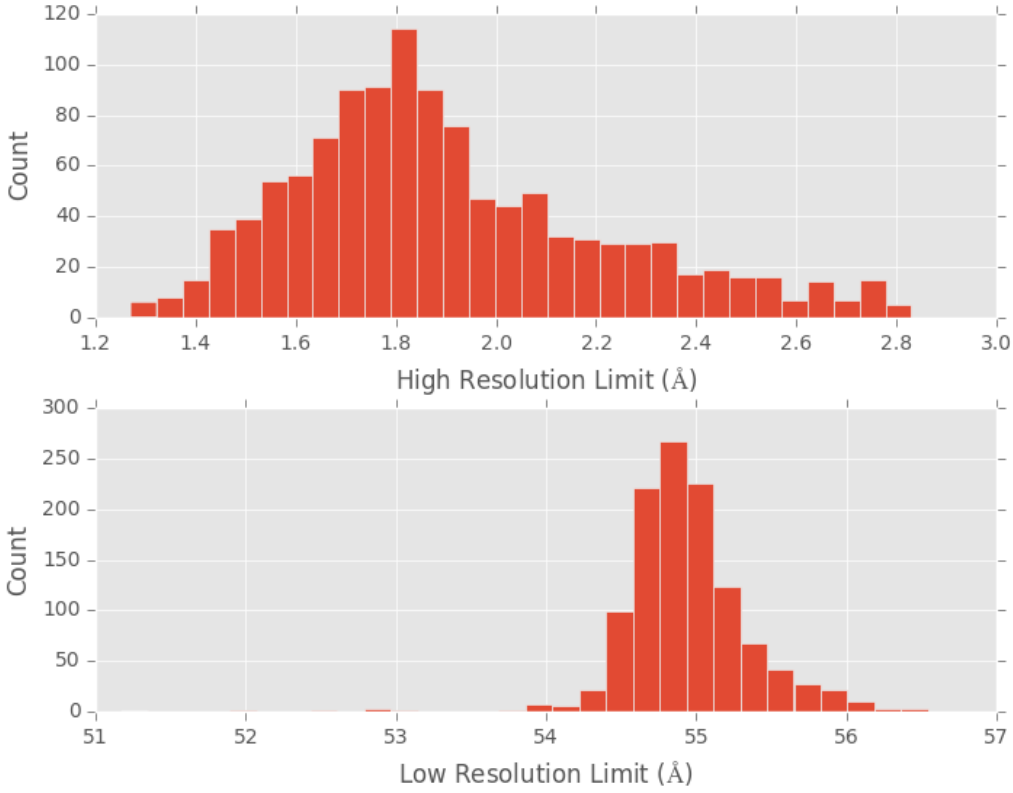

Supplementary Fig. 2. Distribution of resolutions of all datasets analysed by PanDDA.

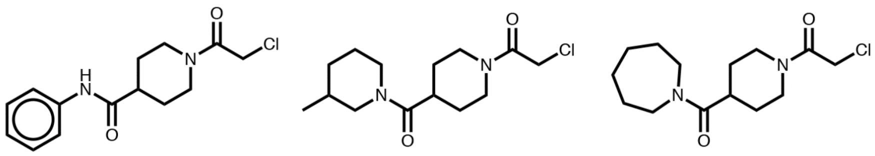

	PCM-0102219	PCM-0102933	PCM-0102781
Labeling	85%	44%	37%
k [$M^{-1}Sec^{-1}$]	$2.80x10^{-7}$	$2.20x10^{-7}$	$1.86x10^{-7}$

Supplementary Fig. 3. Labeling of compounds containing a *N*-chloroacetyl-*N*'-sulfonamido-piperazine or *N*-chloroacetylaniline motifs from electrophile fragment screen.

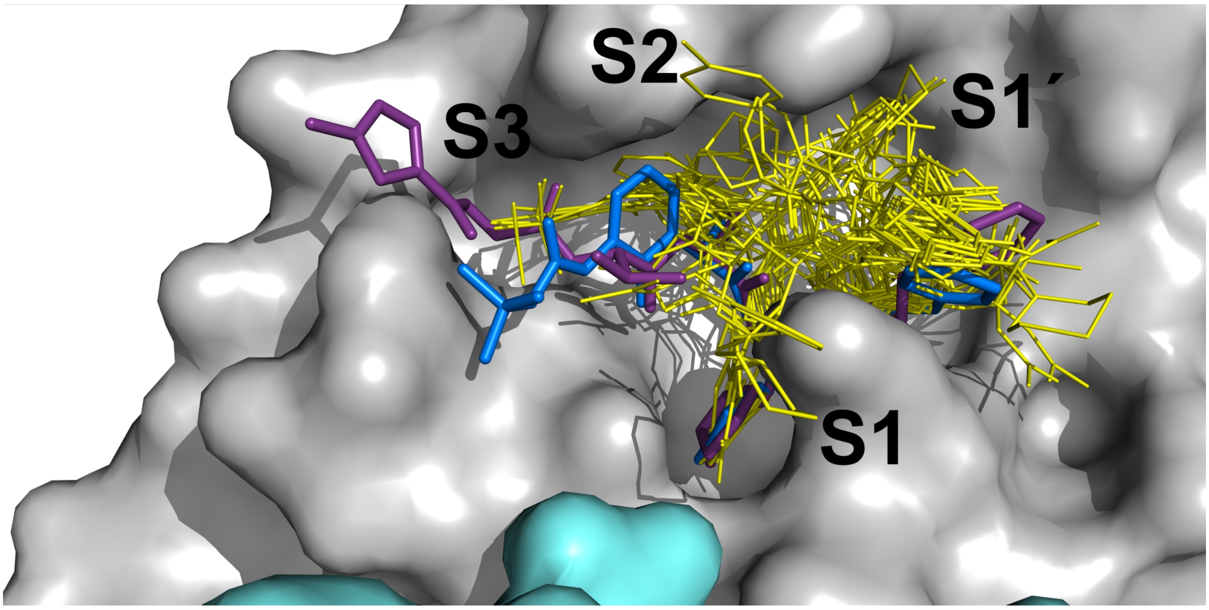

Supplementary Fig. 4. Surface representation of M^pro dimer with fragment hits shown as yellow wires and peptide-based inhibitors from 6LU7 (purple) and 6Y2F (blue) shown as sticks.

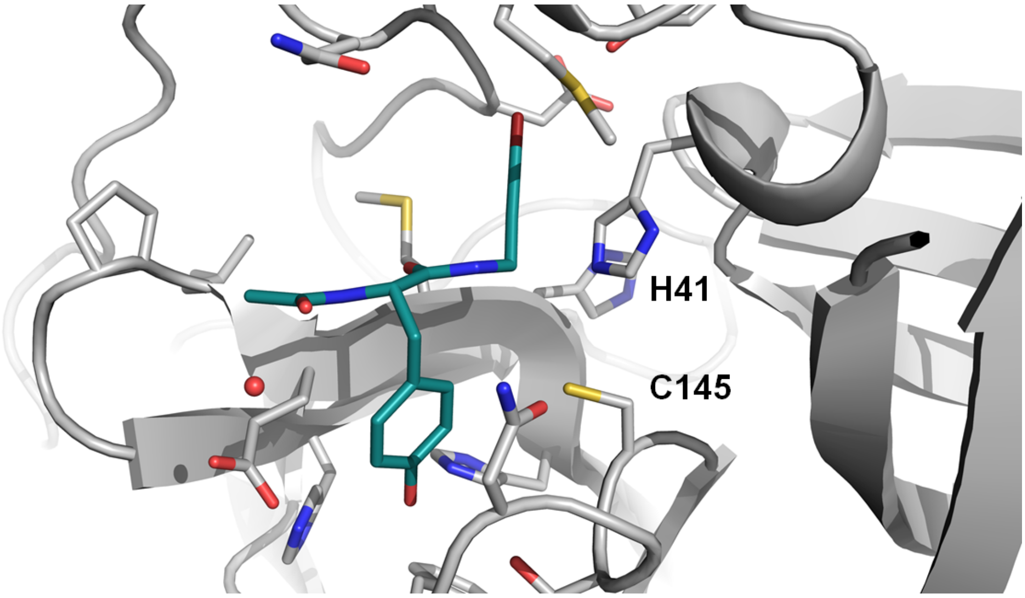

Supplementary Fig. 5. Tyrosine PepLite bound non-covalently in active site.

Supplementary Table 1. Summary of all screened fragments libraries. The table gives an overview of which fragment libraries were screened, how many unique compounds each library contained and finally the number of hits that are currently deposited in the Protein Data Bank.

Fragment Library	Source	Total compounds screened	Number of compounds screened (unique)	Number of successful crystal mounts (unique)	Number of successful data collection (unique)	Number of PDB depositions
DSI poised	Diamond Light Source (UK)	970	695	897	816	39
Fraglites & Peplites	University of Newcastle (UK)	53	53	53	42	4
York3D	University of York (UK)	106	106	102	91	2
MiniFrags	Astex Therapeutics	80	80	60	41	4
electrophile cysteine covalent	Weizmann Institute of Science (Israel)	1084	101	420	381	44
SpotFinder	Hungarian Academy of Sciences	114	96	112	107	1
Heterocyclic electrophilic fragment library	Hungarian Academy of Sciences	474	146	233	160	2

Supplementary Table 2. The expression vector was constructed using a synthetic protease gene, purchased from Integrated DNA Technologies, and In-Fusion cloned into BamHI/XhoI linearised pGEX-6P-1.

Synthetic gene sequence	Vector	Restriction sites
GGGGCCCCTGGGATCCGCTGTCTTACAGTCCGGTTTCCGTAAGATGGCCT<br>TCCCGTCAGGCAAAGTGGAAGGCTGTATGGTGCAAGTAACGTGCGGGACC<br>ACAACGTTGAACGGTTTGTGGTTAGATGATGTGGTTTACTGTCCACGTCA<br>TGTTATCTGTACAAGTGAGGACATGCTTAATCCAAATTATGAGGACTTGT<br>TGATCCGCAAAAGCAACCATAATTTCCTGGTACAGGCAGGCAACGTTCAG<br>TTACGTGTCATTGGTCATTCAATGCAAAACTGCGTGTTGAAGCTGAAAGT<br>AGATACAGCCAACCCGAAAACCCCGAAATATAAATTTGTCCGTATTCAGC<br>CAGGCCAGACCTTTTCGGTTCTGGCGTGCTACAACGGTAGCCCATCTGGG<br>GTCTACCAGTGCGCTATGCGTCCTAACTTTACAATTAAGGGCAGTTTCTT<br>GAACGGTAGCTGCGGAAGCGTTGGCTTTAATATTGACTACGATTGTGTGT<br>CATTTTGCTATATGCACCACATGGAGTTACCCACTGGAGTTCACGCGGGA<br>ACTGACCTGGAGGGGAACTTCTATGGGCCATTTGTGGATCGCCAGACGGC<br>GCAGGCGGCCGGAACGGATACTACTATTACCGTGAATGTCCTTGCTTGGT<br>TATACGCGGCCGTCATTAACGGTGACCGTTGGTTTTTAAACCGTTTCACC<br>ACGACCCTTAATGATTTTAATTTGGTGGCTATGAAGTATAACTACGAACC<br>CCTGACGCAGGACCACGTAGATATTTTGGGGCCGCTGTCGGCACAGACGG<br>GAATTGCAGTGTTAGATATGTGTGCTTCATTGAAAGAGTTGTTACAGAAC<br>GGTATGAATGGACGCACAATTTTGGGATCAGCATTATTAGAGGATGAGTT<br>TACTCCGTTTGATGTTGTGCGTCAGTGCTCGGGTGTAACTTTCCAGGGGC<br>CGCACCATCACCACCATCATTGATCGAGCGGCCGCATC	pGEX-6P-1	BamHI/XhoI

Supplementary References

1 Pearce, N. M. *et al.* A multi-crystal method for extracting obscured crystallographic states from conventionally uninterpretable electron density. *Nat Commun* **8**, (2017).

2 Pearce, N. M., Krojer, T. & von Delft, F. Proper modelling of ligand binding requires an ensemble of bound and unbound states. *Acta Crystallogr D Struct Biol* **73**, 256-266, (2017).
