# Supplementary material for: Crystallographic and electrophilic fragment screening of the SARS-CoV-2 main protease
Source: Nat Commun. 2020 Oct 7;11:5047. doi: 10.1038/s41467-020-18709-w (PMC7542442; doi:10.1038/s41467-020-18709-w)
Supplement: Supplementary file 3 — Description of Additional Supplementary Files [file 41467_2020_18709_MOESM3_ESM.pdf]

Description of Additional Supplementary Files

File Name: Supplementary Data 1
Description: Mass spectrometry fragment screening. 2 µM of Mpro was incubated with a pool of 5 electrophile fragments, 5 µM each at 25°C. After 1.5h the incubation was quenched by adding formic acid at a final concentration of 0.4%.

File Name: Supplementary Data 2
Description: Summary of M^{pro} deposited structures. The table summarises all deposited M^{pro}-fragment structures and how internal crystal IDs map to compound IDs and respective fragment libraries. Furthermore, fragment binding locations are indicated: A – active site, B – active site covalent, C – dimer interface, D – surface, X – crystal contact; residues close to the binding site are shown in parenthesis. The table includes (column F) a subjective assessment of the confidence the crystallographer has in the reliability of the model; these assignments were reviewed by different co-authors.

File Name: Supplementary Data 3
Description: Mass spectrometry fragment screening. 2 µM of Mpro was incubated with 5 µM compound at 25 °C. After 1.5h the incubation was quenched by adding formic acid at a final concentration of 0.4%.

File Name: Supplementary Data 4
Description: Crystallographic Data Collection and Refinement Statistics.
