# Supplementary material for: Crystallographic and electrophilic fragment screening of the SARS-CoV-2 main protease
Source: Nat Commun. 2020 Oct 7;11:5047. doi: 10.1038/s41467-020-18709-w (PMC7542442; doi:10.1038/s41467-020-18709-w)
Supplement: Supplementary file 2 — Peer Review File [file 41467_2020_18709_MOESM2_ESM.pdf]

REVIEWER COMMENTS

Reviewer #1 (Remarks to the Author):

COVID-19 has caught everyone by surprise, but the scientific community appears to have risen as one to find ways to design new therapeutics and re-purpose known clinical drugs. On this note, the current manuscript by Douangamath et al. describes a colossal effort in the fragment-based drug design to screen thousands of compounds, covalent and noncovalent, that bind to the SARS-CoV-2 main protease. The effort is commendable, the manuscript is written clearly, and the data presented are compelling for the next step, which is to merge these fragments, that bind to different substrate subsites, and create a lead inhibitor. The timeline in Figure 2 is very impressive, showing how within 2 months the authors were able to go from protein expression to the deposition of the final structures.
I have several minor comments/suggestions:
1. In the Introduction, a short explanation of the fragment-based drug design methodology and its advantages need to be articulated for a non-initiated reader. Also, mention whether or not this technology was used in the design of SARS-CoV-1 inhibitors, and connect all this info to the fact that no clinical drugs have appeared from the 17-year research efforts into SARS-CoV-1.
2. In the subsection named "Dimer interface fragments", mention that those fragments bind to exposed areas in the dimer interface and do not actually disrupt the enzyme dimerization.
3. Line 194, change "sample" to "substrate".
4. Paragraph starting on line 302. It is important to make a clear distinction between reversible and irreversible inhibitors. Most of the compounds found in the current study are irreversible ligands, because the final product is the thioether, whereas ligands with aldehyde or ketone groups are reversible inhibitors. This can be mentioned directly in this paragraph.
5. Comment on the low occupancy (<40%) of many ligands in the reported structures. Accuracy in the atomic positions for such ligands is low, complicating structural analysis. Also, for some data CC1/2 values in the highest resolution shell are very low (~0.1). Is it justified to use data to such resolutions?
6. Provide CC* values for the unliganded structure 6YB7 in Table S4.

Reviewer #2 (Remarks to the Author):

The manuscript entitled "Crystallographic and electrophilic fragment screening of the SARS-1 CoV-2 main protease" provides a valuable contribution to the development of therapeutic agents against SARS-CoV-2. The authors identified 74 fragment hits bound to the main viral protease (Mpro) which can be used to design more potent compounds with improved binding properties. The described approach, which combines electrophilic and crystallographic screening, reveals in addition active site plasticity, what renders in silico screening approaches much less attractive and unreliable.
Importantly, the presented results were publicly available before all the experiments have been completed and described in the form of a manuscript. However, at present, structures (most of them) are not available from the PDB, although atomic models can be downloaded via https://www.ncbi.nlm.nih.gov/Structure/pdb/ web interface.

The manuscript merits publication as it provides a significant contribution not only to the development of an antiviral drug against SARS-CoV-2 but also to the development of probe molecules with the aim of understanding viral biology. Despite it, some minor concerns should be clarified.

Remarks:

The authors have used several automated/semi-automated programs for data processing xia2,

autoPROC and DIALS employing XDS. What was the reason to exploit different data processing pipelines? I think it could be of interest for others, most likely less experienced people doing the crystallographic screening, to be provided with more details explaining whether default settings or customized ones have been used.

Some data sets reveal quite high R-merge, Rmeas and R-pim values (more than 15% overall and more than 100% in the highest resolution shell) with the overall redundancy below 4. Despite the fact that CC(1/2) is still acceptable (> 0.3 for the highest resolution shell) it is surprising that some ligands have been classified as "4 – High Confidence" (e.g.: 5RFN) although in case of a structure of similar quality (5RFM) the ligand molecule has been classified as "2 – Correct ligand, weak density". This discrepancy could be explained by ligand occupancy (0.72 vs. 0.50) but is not reflected in reported ligand CC (0.8 vs. 0.87). I am aware that correlation coefficient could be misleading in case of a noisy electron density map, but this raises the question of how reliable are results obtained from some data sets? How was the ligand CC (listed in Table S4) calculated and against which electron density map? Was it the PanDDA's event map or standard 2mFo-DFc map calculated for the final structure including the ligand molecule?

Were diffraction images manually inspected in order to identify problems leading to elevated R-merge, Rmeas and R-pim values? Did the authors consider excluding some data sets from the analysis due to a lower quality of diffraction data or limited interpretability of the calculated electron density map resulting from lower ligand occupancy and/or higher positional disorder (elevated ADPs)? Or just all data sets have been processed by PanDDA?

Details describing crystallographic refinement strategy (Table S4) are missing, e.g.: isotropic or anisotropic B-factor refinement, TLS, final model refined with Buster/Refmac5.

We were pleased to receive the very positive review comments for our paper "**Crystallographic and electrophilic fragment screening of the SARS-CoV-2 main protease**". Below please find our point by point response to the reviewer comments

Reviewer #1

1. In the Introduction, a short explanation of the fragment-based drug design methodology and its advantages need to be articulated for a non-initiated reader. Also, mention whether or not this technology was used in the design of SARS-CoV-1 inhibitors, and connect all this info to the fact that no clinical drugs have appeared from the 17-year research efforts into SARS-CoV-1.

We reworked part of the introduction to address these themes (highlighted yellow text lines 39-70).

2. In the subsection named "Dimer interface fragments", mention that those fragments bind to exposed areas in the dimer interface and do not actually disrupt the enzyme dimerization.

Text (line 192-193) has been modified from "In this study three compounds bound at the dimer interface." to "In this study three compounds bound at accessible sites of the dimer interface, that conceivably could be exploited to design compounds to disrupt the M^{Pro} dimer."

3. Line 194, change "sample" to "substrate".

Noted and changed (now on line 212)

4. Paragraph starting on line 302. It is important to make a clear distinction between reversible and irreversible inhibitors. Most of the compounds found in the current study are irreversible ligands, because the final product is the thioether, whereas ligands with aldehyde or ketone groups are reversible inhibitors. This can be mentioned directly in this paragraph.

The following text has been added (lines 347-350):
"We note here that most of the electrophiles that we screened (chloroacetamides and acrylamides) form irreversible adducts with the target cysteine, whereas many protease inhibitors contain aldehydes, nitriles and α-ketoamides, that can form reversible covalent bonds."
*To give the reader further context elsewhere, we have also added the following sentence (and 5 references) at the beginning of the section "*Combined MS and crystallographic fragment screens reveal new binders of M^{pro}" *(lines 112-113)*: *"…, and screening covalent fragments is known to be useful at identifying effective starting points." (text highlighted in yellow)*

5. Comment on the low occupancy (<40%) of many ligands in the reported structures. Accuracy in the atomic positions for such ligands is low, complicating structural analysis. Also, for some data CC1/2 values in the highest resolution shell are very low (~0.1). Is it justified to use data to such resolutions?

These are indeed regular questions for such experiments, and the reviewer is correct to point out that the methodology is not yet old enough for it not to require explanation. We have included an additional paragraph in the discussion (lines 311-24), specifically explaining why both these observations do not affect the reliability of the models.
We note here (but not in the text) that the majority of the data sets have $CC_{1/2}$ values around 0.3 or greater, but as noted by the referee two data sets have much lower $CC_{1/2}$ values than the default cutoff of 0.3 (0.106 for Mpro-x0165/5RGG; 0.158 for Mpro-x0749/5REN). The diffraction data for these is of poor quality however, in these cases the CC values, which provides a statistic to directly compare model quality and data quality, are 0.44 and 0.52 respectively. The PanDDA event maps are of high quality (shown below) and allowed modelling of the expected fragments. Hence, we believe that it was justified to use and include data*

to such resolutions for this use case. We have also updated the supplementary S1 section to further clarify estimation of ligand occupancy with the following text:

"Ligand occupancy is initially estimated by PanDDA using the Background Density Correction factor (BDC) that maximises the density signal[1], but the final occupancy comes from refinement."

Furthermore, in supplementary S1 section we have also added the following which directs the reader to a web server that allows an easy to use online webform for inspection of the experimental electron density maps of all the deposited structures:

"The electron density evidence was made available in a generally accessible format online, at

https://www.diamond.ac.uk/covid-19/for-scientists/Main-protease-structure-and-XChem/Electron-density-evidence.html. "

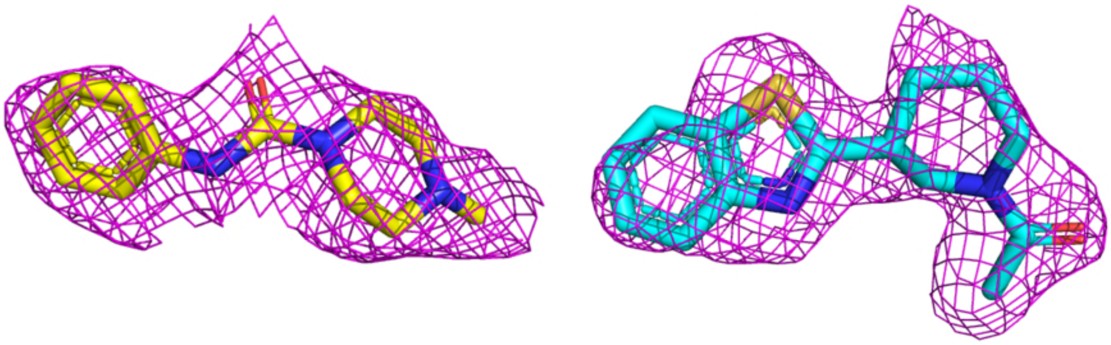

PanDDA event maps contoured at 1 σ and carved at 1.6 Å around modelled ligand from deposited pdbs Left: X0165/5RGG. Right: X0749/5REN

6. Provide CC* values for the unliganded structure 6YB7 in Table S4.

CC values for 6YB7 have been updated in table S4.*

Reviewer #2

1. Importantly, the presented results were publicly available before all the experiments have been completed and described in the form of a manuscript. However, at present, structures (most of them) are not available from the PDB, although atomic models can be downloaded via https://www.ncbi.nlm.nih.gov/Structure/pdb/ web interface.

We have checked all PDB entries in the supplementary information table S4, and can confirm that 92 of these were released before the 15th April 2020, and the remaining 5 entries on the 10th June 2020.

2. The authors have used several automated/semi-automated programs for data processing xia2, autoPROC and DIALS employing XDS. What was the reason to exploit different data processing pipelines? I think it could be of interest for others, most likely less experienced people doing the crystallographic screening, to be provided with more details explaining whether default settings or customized ones have been used.

The automated data reduction pipelines are run by default using all the main data reduction software packages allowing the user ultimately to select the data to take forward. All processing is performed using standard input. We have revised the methods section (lines 437-448, highlighted in yellow) with background references to direct reader as suggested by referee.

"Data were collected at the beamline I04-1 at 100K and processed with the fully automated pipelines at Diamond[64,72,73], which variously combine XDS[74], xia2[65], autoPROC[75] and DIALS[64], and select resolution limits algorithmically; no manual curation of processing parameters was applied. Further analysis was performed through XChemExplorer[27]: for each dataset, the version of processed data was selected by the default XChemExplorer score, and electron density maps were generated with Dimple[76]. Ligand-binding events were identified using PanDDA[37] (both the released version 0.2 and a pre-release development version (https://github.com/ConorFWild/pandda)), and ligands were modelled into PanDDA-calculated event maps using Coot[67]. Restraints were calculated with ACEDRG or GRADE[77,78], structures were refined with Refmac[68] and Buster[69], and models and quality annotations cross-reviewed . Further elaboration of the PanDDA analysis is provided in the supplementary information (S1)."

3. Some data sets reveal quite high R-merge, Rmeas and R-pim values (more than 15% overall and more than 100% in the highest resolution shell) with the overall redundancy below 4. Despite the fact that CC(1/2) is still acceptable (> 0.3 for the highest resolution shell) it is surprising that some ligands have been classified as "4 – High Confidence" (e.g.: 5RFN) although in case of a structure of similar quality (5RFM) the ligand molecule has been classified as "2 – Correct ligand, weak density". This discrepancy could be explained by ligand occupancy (0.72 vs. 0.50) but is not reflected in reported ligand CC (0.8 vs. 0.87). I am aware that correlation coefficient could be misleading in case of a noisy electron density map, but this raises the question of how reliable are results obtained from some data sets? How was the ligand CC (listed in Table S4) calculated and against which electron density map? Was it the PanDDA's event map or standard 2mFo-DFc map calculated for the final structure including the ligand molecule?

In the case of the classification of the confidence of ligand placement, this is a qualitative assessment based on user judgement of the corresponding PanDDA event map. However, ligand CC was calculated against the final 2mFo-DFc map with the ligand included in the model and the values are often significantly lower than what is typically accepted. However, we have double-checked 5RFN and 5RFM and 5RFN, which has a subjectively higher ligand confidence, has also a higher ligand CC (0.87), whereas 5RFM, which has lower ligand confidence, has also a lower ligand CC (0.80). Unfortunately, ligand placement, even when supported by the PanDDA method is still often subjective. Moreover, occupancy refinement may not always be entirely robust. However, given that the algorithm has no information about soaked or co-crystallised ligands, and the resulting event maps, even for weak ligands, still showed recognisably the shape of the added small molecule, we felt it is important to make this information accessible.

These points are addressed in the same new paragraph (lines 311-24) and via the updated supplementary S1 section that addresses comment 5 of reviewer #1.

4. Were diffraction images manually inspected in order to identify problems leading to elevated R-merge, Rmeas and R-pim values? Did the authors consider excluding some data sets from the analysis due to a lower quality of diffraction data or limited interpretability of the calculated electron density map resulting from lower ligand occupancy and/or higher positional disorder (elevated ADPs)? Or just all data sets have been processed by PanDDA?

This is addressed in the same new paragraph (lines 311-24) addressing point 5 of reviewer #1, we have also updated the methods section with the modified text(lines 437-448) as detailed in point 2 of reviewer #2 to clarify the data processing workflow

5. Details describing crystallographic refinement strategy (Table S4) are missing, e.g.: isotropic or anisotropic B-factor refinement, TLS, final model refined with Buster/Refmac5.

Table S4 We have added a row to state what program was used in refinement (Refmac or Buster) and added a footnote to summarise the refinement strategies used:
"..where Refmac was used, models were refined as composite models comprising both the ligand-bound and confounding ground states, as described previously [Pearce et al, ActaD, 2017]. In models refined with Buster, the bound state alone was placed, and included NCS restraints and TLS parameters. In addition we have added a footnote to state the ligand CC was calculated against the final 2mFo-DFc map from refinement with the ligand included in the model
We have also provided an alternative vertical view of the table in addition to the more traditional view for such statistics

REVIEWERS' COMMENTS:

Reviewer #2 (Remarks to the Author):

The revised manuscript entitled “Crystallographic and electrophilic fragment screening of the SARS-1 CoV-2 main protease” provides missing details and explanations, which satisfactorily addressed my comments and questions. I am convinced that upon uploading the proper file for Table S2 the manuscript will merit publication.
